# CK2B Induces CD8^+^ T‐Cell Exhaustion through HDAC8‐Mediated Epigenetic Reprogramming to Limit the Efficacy of Anti‐PD‐1 Therapy in Non‐Small‐Cell Lung Cancer

**DOI:** 10.1002/advs.202411053

**Published:** 2025-02-27

**Authors:** Shaochuan Liu, Shiya Ma, Gen Liu, Lingjie Hou, Yong Guan, Liang Liu, Yuan Meng, Wenwen Yu, Ting Liu, Li Zhou, Zhiyong Yuan, Shuju Pang, Siyuan Zhang, Junyi Li, Xiubao Ren, Qian Sun

**Affiliations:** ^1^ Tianjin Medical University Cancer Institute and Hospital National Clinical Research Center for Cancer Tianjin 300060 China; ^2^ Key Laboratory of Cancer Prevention and Therapy Tianjin 300060 China; ^3^ Tianjin's Clinical Research Center for Cancer Tianjin 300060 China; ^4^ Key Laboratory of Cancer Immunology and Biotherapy Tianjin 300060 China; ^5^ Department of Immunology Tianjin Medical University Cancer Institute and Hospital, Tianjin Medical University Tianjin 300060 China; ^6^ Department of Radiation Oncology Tianjin Medical University Cancer Institute and Hospital, Tianjin Medical University Tianjin 300060 China; ^7^ Department of Biotherapy Tianjin Medical University Cancer Institute and Hospital, Tianjin Medical University Tianjin 300060 China; ^8^ Department of Radiation Oncology Chongqing University Cancer Hospital Chongqing 400030 China

**Keywords:** CK2B, ICIs, NSCLC, T cell exhaustion, TME

## Abstract

Anti‐PD‐1 therapy has left an indelible mark in the field of non‐small‐cell lung cancer (NSCLC) treatment; however, its efficacy is limited in clinical practice owing to differences in the degree of effector T‐cell exhaustion. Casein kinase 2 (CK2) is a protein kinase that plays an important role in T‐cell immunity. In this study, it is aimed to explore the potential of targeting CK2 and its regulatory subunit CK2B to prevent or reverse T‐cell exhaustion, thereby enhancing the efficacy of anti‐PD‐1 therapy in NSCLC. In this study, it is found that CK2B expression is closely associated with T‐cell exhaustion as well as the efficacy of anti‐PD‐1 therapy based on scRNA‐seq and in vitro and in vivo experiments. Utilization of CK2 inhibitors or knockdown of CK2B expression can upregulate TBX21 expression through HDAC8‐mediated epigenetic reprogramming, restoring the effector function of CD8^+^ T cells and enhancing the efficacy of anti‐PD‐1 therapy in NSCLC. These findings underscore CK2B as a promising target for overcoming the exhaustion of effector CD8^+^ T cells, thereby enhancing the efficacy of anti‐PD‐1 and adoptive cell therapies in NSCLC. Moreover, CK2B expression serves as a novel predictor of immunotherapy efficacy for NSCLC.

## Graphical abstract

αPD‐1 therapy has left an indelible mark in the field of NSCLC treatment; however, its efficacy is limited in clinical practice owing to the effector T‐cell exhaustion. These findings underscore CK2B as a promising target for overcoming the exhaustion of effector CD8^+^ T cells, thereby enhancing the efficacy of αPD‐1 and adoptive cell therapies in NSCLC.

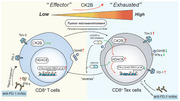

## 1 Introduction

Despite significant advances in radiotherapy, targeted therapy and immunotherapy, non‐small‐cell lung cancer (NSCLC) remains one of the leading causes of cancer‐related death. The advent of immune checkpoint inhibitors (ICIs) has dramatically shifted the paradigm of cancer therapy; however, only a few patients with NSCLC have been able to achieve durable clinical benefit from this therapy.^[^ ^1^, ^2^, ^3^ ^]^ T‐cell exhaustion within the tumor microenvironment (TME) is a critical factor in mediating resistance to ICIs.^[^ ^4^, ^5^ ^]^

T‐cell exhaustion represents a distinct state of the immune system characterized by the progressive loss of T‐cell function owing to sustained antigenic stimulation.^[^ ^6^ ^]^ This state encompasses a spectrum ranging from precursor‐exhausted T cells (Tpex), which retain stem cell‐like proliferative properties, to terminally exhausted T cells (Tex‐term), which have lost their effector function and proliferative potential. Notably, TCF‐1^+^CD8^+^ Tpex are transiently activated and undergo significant expansion during anti‐PD‐1 therapy; the degree of expansion serves as a predictor of immunotherapy efficacy.^[^ ^7^, ^8^, ^9^ ^]^ However, despite blocking the PD‐1/PD‐L1 pathway, these transiently activated Tpex cells fail to undergo epigenetic reprogramming and differentiate into Tex‐term cells that are unresponsive to ICIs. This process ultimately contributes to resistance to ICI therapies.^[^ ^9^, ^10^ ^]^

Casein kinase 2 (CK2), a serine/threonine kinase, forms a tetrameric structure primarily comprising two catalytic subunits (CK2α and/or CK2α') and two regulatory subunits (CK2β).^[^ ^11^ ^]^ Different subunits are encoded by separate genes, such as *CSKN2A1*, *CSKN2A2*, and *CSNK2B*, and each subunit can independently exert effector functions autonomously from the tetramer.^[^ ^12^ ^]^ CK2 phosphorylates over 500 substrates, accounting for approximately 10% of the phosphorylated proteome in humans.^[^ ^13^ ^]^ Many CK2 substrates play critical roles in cell development, differentiation, epigenetic regulation, and DNA damage repair through a variety of pathways (e.g., PI3K/AKT, NFKB, JAK/STAT3, and WNT).^[^ ^14^ ^]^

Knockdown of CK2B or application of CK2 inhibitors inhibits the phosphorylation of STAT3 in CD4^+^ T cells, subsequently altering their differentiation into Tregs and Th17 cells.^[^ ^15^ ^]^ Additionally, CK2 expression and activity are consistently upregulated during CD8^+^ T‐cell stimulation and play a crucial role in regulating effector functions.^[^ ^16^ ^]^ Although sustained antigenic stimulation is closely associated with T‐cell exhaustion, the involvement of CK2 in the regulation of T‐cell exhaustion remains unclear.

Here, we aimed to examine the role of CK2B in T‐cell exhaustion during resistance to anti‐PD‐1 therapy in NSCLC using scRNA‐seq and in vitro and in vivo experiments. Furthermore, we aimed to elucidate the mechanism by which CK2B regulates T‐cell exhaustion and explore whether CK2B could serve as a potential target for intervening in the process of T‐cell exhaustion in immunotherapy for NSCLC and enhancing the efficacy of immunotherapy.

## 2 Results

### 2.1 High CSNK2B Expression in CD8+ T Cells is Closely Associated with Poor Pathological Remission Rates and T‐Cell Exhaustion

**Figure 1:**
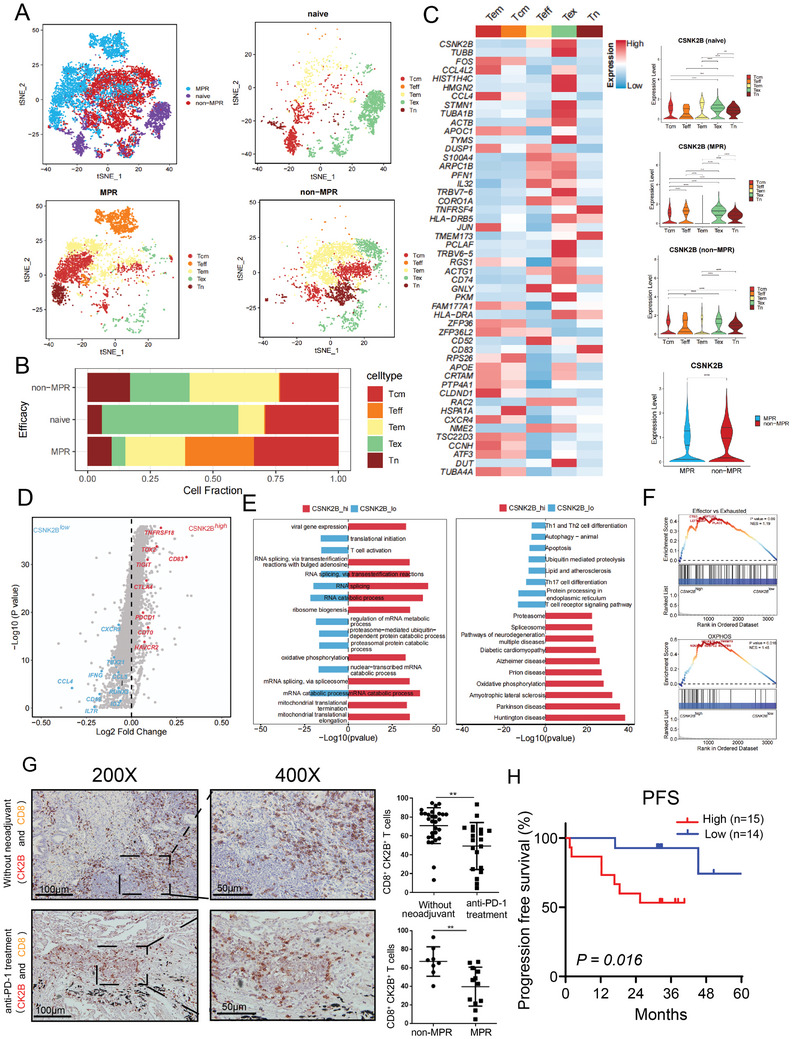
High expression of *CSNK2B* in CD8**^+^** T cells of tumor sites from patients with NSCLC is associated with poor prognosis. A) tSNE clustering analysis of CD8^+^ T‐cell single‐cell sequencing data from tumor tissues of 12 patients with NSCLC in naïve group, MPR group, and Non‐MPR group. B) Proportion of each subset of CD8^+^ T cells in naïve, Non‐MPR, and MPR groups. C) Differential gene clustering analysis of each CD8^+^ T‐cell subpopulation (red indicates high expression, blue indicates low expression) and statistical plots of *CSNK2B* expression. D) Volcano plot showing differential genes between two groups of *CSNK2B* ^high^ *CD8* ^+^ T cells and *CSNK2B* ^low^ *CD8* ^+^ T cells, with red representing genes highly expressed in the *CSNK2B* ^high^ *CD8* ^+^ T‐cell group and blue representing genes highly expressed in the *CSNK2B* ^low^ *CD8* ^+^ T‐cell group. E) GO and KEGG pathway analyses. F) Gene set enrichment analysis was performed to determine the specific enrichment in T cell exhaustion and oxidative phosphorylation‐related gene signatures among *CSNK2B* ^high^ *CD8* ^+^ T‐cells and *CSNK2B* ^low^ *CD8* ^+^ T‐cells. G) Immunohistochemical double staining labeling CK2B (red) and CD8 (brown), scale = 50 or 100 µm. H) Correlation between high and low CK2B expression in tumor‐infiltrating CD8^+^ T cells and progression‐free survival in patients with NSCLC. *P < 0.05, **P < 0.01, ***P < 0.001, and ****P < 0.0001 (log rank test or Student's t test).

Our work was performed by collecting 10127 CD8^+^ tumor‐infiltrating lymphocytes (TILs) from 12 NSCLC patients for single‐cell analysis (4 patients without anti‐PD‐1 neoadjuvant therapy [naïve], 4 in the major pathologic response [MPR] group after receiving anti‐PD‐1 therapy, and 4 patients that did not achieve major pathologic remission [Non‐MPR] after receiving anti‐PD‐1 therapy). In the Non‐MPR group, a higher percentage of CD8^+^ Tex cells and a lower ratio of CD8^+^ effector T cells were observed compared to the MPR group (**Figure** **1**A,B).

We further analyzed differential gene expression across various CD8^+^ T cell subpopulations, identifying the top 50 genes with the most significant expression changes. Specifically, we compared CD8^+^ Tex cells with other T cell populations, including naïve T cells (Tn), central memory T cells, effector memory T cells, and effector T cells (Teff). This analysis highlighted genes such as *CSNK2B*, *PKM*, and *PCLAF* as the most significantly differentially expressed (Figure 1C). Furthermore, we tested the consistency of these significantly upregulated genes in public databases, with *CSNK2B* showing the most significant differential expression. In addition, CD8^+^ T cells in the non‐MPR group showed higher expression of *CSNK2B* than those in the MPR group (*P < *0.0001) (Figure 1C). The above findings suggest that *CSNK2B* expression is closely related to the exhaustion of CD8^+^ T cells and the efficacy of anti‐PD‐1 therapy.

Subsequently, we divided CD8^+^ TILs from NSCLC patients undergoing anti‐PD‐1 therapy into *CD8* ^+^ *CSNK2B* ^high^ and *CD8* ^+^ *CSNK2B* ^low^ groups, according to the level of *CSNK2B* expression. We found that genes encoding immunosuppressive checkpoint‐associated (*PDCD1*, *TIGIT*, *CTLA4*, *CD70*, and *HAVCR2*) and exhaustion‐associated (*TOX2* and *TNFRSF18*) transcripts were highly expressed in the *CD8* ^+^ *CSNK2B* ^high^ group (Figure 1D). By contrast, genes related to the activation and capability of T cells (*CCL4*, *IFNG*, *CCL5*, *TBX21*, etc.) were highly strongly in the *CD8* ^+^ *CSNK2B* ^low^ group (Figure 1D).

We also performed KEGG and GO analyses of the genes with differential expression from *CD8* ^+^ *CSNK2B* ^low^ group and *CD8* ^+^ *CSNK2B* ^high^ group, and found that pathways related to oxidative phosphorylation were enriched strongly in the *CD8* ^+^ *CSNK2B* ^high^ group, whereas those related to T‐cell activation and endoplasmic reticulum (ER) were enriched strongly in the *CD8* ^+^ *CSNK2B* ^low^ group (Figure 1E). In addition, GSEA of transcriptional differences between the *CD8* ^+^ *CSNK2B* ^high^ and *CD8* ^+^ *CSNK2B* ^low^ groups showed a close correlation between gene sets and pathways associated with T‐cell oxidative phosphorylation (NES = 1.45) and exhaustion (NES = 1.19) (Figure 1F). Moreover, trajectory analysis revealed that *CSNK2B* expression in CD8^+^ Tn cells increased gradually during their differentiation into Tex cells (Figure S1A, Supporting Information). We also assessed the expression of other CK2 subunits in CD8^+^ Tex cells, and found that neither *CSNK2A1* nor *CSNK2A2* was increased significantly during CD8^+^ T‐cell differentiation in the naïve group (Figure S1B, Supporting Information). Their expression levels remained relatively low compared to *CSNK2B*, with a relative expression value of less than 1.0 (Figure S1B, Supporting Information).

A similar phenomenon was observed in public databases (http://lung.cancer‐pku.cn/index.php), where the expression levels of *CSNK2A1* and *CSNK2A2* were low in CD8^+^ Tex cells and not significantly upregulated compared to other cell populations, except for *CSNK2B* (Figure S1C, Supporting Information). Similar findings were observed in a model of induced T cell exhaustion, where the mRNA expression level of *CSNK2B* was significantly higher compared to *CSNK2A1* and *CSNK2A2* in CD8^+^ Tex cells (Figure S1D, Supporting Information). Furthermore, we also observed that the expression levels of CSNK2A1 and CSNK2A2 did not show a significant increase over time, from 0 to 72 h in the induced exhaustion model (Figure S1E, Supporting Information). In summary, the above results suggest that CK2B expression in CD8^+^ T cells from NSCLC patients is favorably correlated with T cell exhaustion and unfavorably correlated with the efficacy of anti‐PD‐1 therapy.

We also found no significant difference in *CSNK2B* expression between normal and tumor tissues [including lung adenocarcinoma (LUAD) and lung squamous cell carcinoma (LUSC)] upon analyzing the GEPIA public database. Furthermore, there was no meaningful association between *CSNK2B* expression and prognosis in NSCLC patients (Figure S2A,B, Supporting Information). This suggests that *CSNK2B* expression in tumor tissues does not significantly affect the survival of patients with NSCLC. Since tumor tissues predominantly consisted of tumor cells, any potential impact of CSNK2B expression specifically in NSCLC tumor cells on patient prognosis was eliminated.

Furthermore, we analyzed scRNA‐seq data (GSE99254) and found that *CSNK2B* was strongly expressed in CD8^+^ Tex cells in contrast to non‐Tex cells, and favorably association with *HAVCR2* and *CTLA‐4* expression (Figure S2C, Supporting Information). This result corroborated our previous finding that *CSNK2B* expression was favorably association with the exhaustion of CD8^+^ T cells.

Next, we collected paraffin tissue sections of patients with NSCLC undergoing anti‐PD‐1 neoadjuvant therapy or not for immunohistochemical double staining. We found that patients who responded effectively to anti‐PD‐1 neoadjuvant therapy in the MPR group exhibited fewer CD8^+^CK2B^+^ T cells compared to those in the non‐MPR group (*P *< 0.01) (Figure 1G). Moreover, patients receiving anti‐PD‐1 neoadjuvant therapy had fewer tumor‐infiltrating CD8^+^CK2B^+^ T cells than those who did not receive neoadjuvant therapy (*P *< 0.01) (Figure 1G). This result also suggests that anti‐PD‐1 therapy promotes the expansion of Teff cells, influencing the expression of CK2B. We also found that high CK2B expression in CD8^+^ T cells from patients with NSCLC not receiving neoadjuvant therapy predicted poor progression‐free survival (PFS) (*P* = 0.016) (Figure 1H). These results demonstrate that CK2B expression in tumor cells did not affect patient prognosis. By contrast, CK2B expression in CD8^+^ T cells was significantly associated with the efficacy of anti‐PD‐1 therapy and survival in patients with NSCLC.

### 2.2 Inhibition of CK2 or Knockdown of CK2B Expression could Prevent the Exhaustion Process of CD8^+^ T Cells In Vitro

Figure 1E shows that CK2B expression is closely related to the ER‐related pathways; therefore, we further explored whether ER stress induces CK2B expression. According to a previous study,^[^ ^17^ ^]^ αCD3/CD28 mAbs was used to induce ER‐stress in T cells. GRP78, which is an indicator of the degree of ER‐stress,^[^ ^18^ ^]^ and CK2B, were upregulated during ER stress induction (Figure S3A, Supporting Information). We observed a similar phenomenon in the mouse CD8^+^ T cells (Figure S3B, Supporting Information). Our experiments revealed that stimulating ER stress (thapsigargin, Th) led to an increase in CK2B expression, both in human and mouse CD8^+^ T cells. Conversely, inhibiting ER stress (tauroursodeoxycholic, TD) showed the opposite effect, suggesting a reciprocal regulation between ER stress levels and CK2B expression in these immune cells (Figure S3C,D, Supporting Information). In addition, we collected the supernatant of human A549 cells prepared as a tumor‐conditioned medium and used it to stimulate CD8^+^ T cells. We found that the tumor‐conditioned medium up‐regulated CK2B expression in CD8^+^ T cells (Figure S3E, Supporting Information). At the mRNA level, we found that the application of thapsigargin significantly increased *CSNK2B* expression, as well as genes associated with ER‐stress such as *XBP1* and *DDIT3*, whereas the expression of the related mRNAs was downregulated by the application of tauroursodeoxycholic acid (Figure S3F, Supporting Information). The above results suggest that ER stress regulates CK2B expression in CD8^+^ T cells.

Reactive oxygen species^[^ ^19^ ^]^(ROS) are important factors in inducing ER stress, and we assessed ROS expression levels in human and mouse CD8^+^ T cells using flow cytometry following 24 h of induction with tumor‐conditioned medium. We found that ROS expression levels were significantly increased by stimulation with tumor‐conditioned medium (Figure S3G, Supporting Information). The above results suggest that raised ROS levels in CD8^+^ T cells when stimulated by tumor‐conditioned medium leads to heightened ER‐stress, thereby augmenting the expression of CK2B.

**Figure 2:**
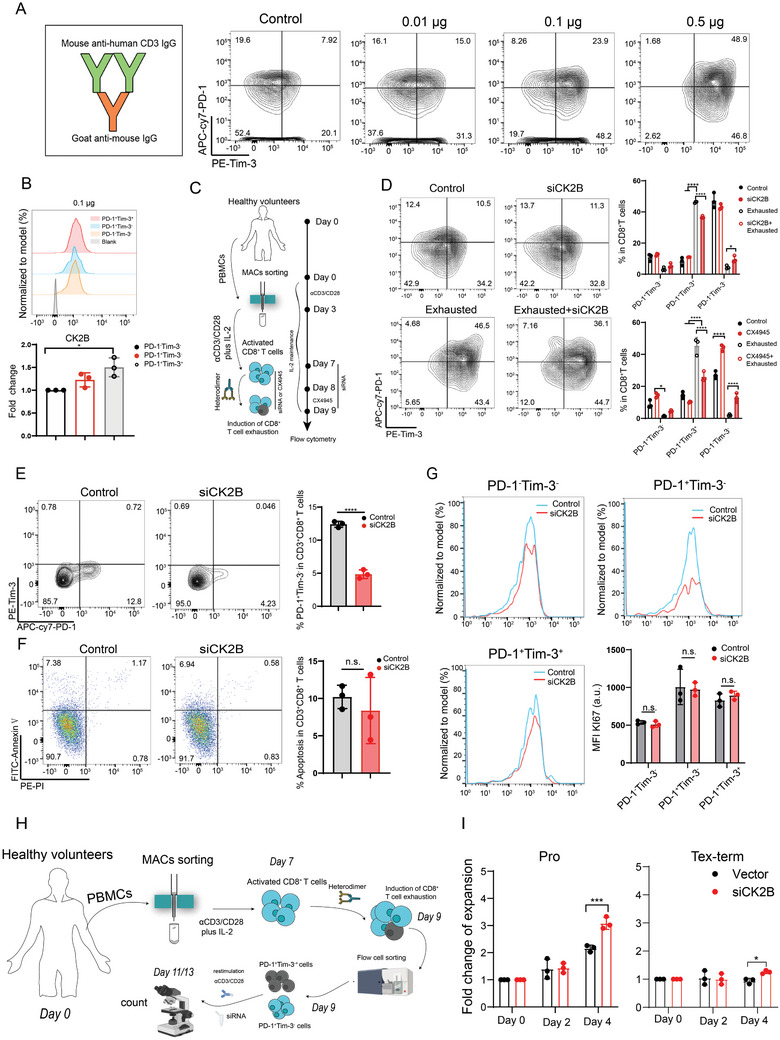
CK2B regulates the exhaustion process of CD8^+^ T cells in vitro. A) Schematic experimental setup of the establishment of a CD8^+^ T‐cell exhaustion model in vitro. B) Representative flow cytometry plots (upper) and fold change (bottom) showing CK2B expression on three subpopulations of CD8^+^ T cells from a CD8^+^ T‐cell exhaustion model (heterodimers were treated at a concentration of 1 µg mL^−1^ for 48 h). C) Schematic experimental setup of CX4945 and siRNA intervention during induction of T‐cell exhaustion. D) PD‐1 and Tim‐3 expression levels in CD8^+^ T cells after treatment with siRNA or CX4945 (10 µM) were detected by flow cytometry. E) Flow cytometry analysis of the percentage of PD‐1^+^Tim‐3^−^CD8^+^ T cells in CD8^+^ T cells after treatment with siRNA for 48 h. F) Effect of siRNA treatment for 48 h on CD8^+^ T‐cell apoptosis (early apoptosis). G) Effect of siRNA treatment for 48 h on Ki67 expression of various CD8^+^ T‐cell subpopulations. H) The schematic shows the expansion experiments of PD‐1^+^Tim‐3^−^CD8^+^ T cells or PD‐1^+^Tim‐3^+^CD8^+^ T cells activated by re‐stimulation after siRNA treatment. I) The statistical plots show the fold change of expansion of PD‐1^+^Tim‐3^−^CD8^+^ T cells and PD‐1^+^Tim‐3^+^CD8^+^ T cells recorded by microscopy on days 2 and 4 after re‐αCD3/CD28 stimulation. Exhausted: heterodimers; Pro: Tpex; Tex‐term: terminally exhausted T cells; *P < 0.05, **P < 0.01, ***P < 0.001, and ****P < 0.0001 (Student's t test); n.s., not significant (*P* > 0.05).

After investigating the factors influencing CK2B expression, we further explored the impact of increased CK2B expression on T cell exhaustion. Therefore, we established a model for inducing the exhaustion of CD8^+^ T cells according to previous research^[^ ^20^, ^21^ ^]^ (**Figure** **2**A). According to previous literature,^[^ ^21^ ^]^ PD‐1^+^Tim‐3^+^TCF‐1^−^ T cells are recognized as Tex‐term cells, and PD‐1^+^Tim‐3^−^TCF‐1^+^ T cells are classified as Tpex cells. Our experiments similarly found that PD‐1^+^Tim‐3^+^ T cells lack expression of TCF‐1, and PD‐1^+^Tim‐3^−^ T cells show positive TCF‐1 expression (Figure S4A, Supporting Information). Building on this observation, we differentiated between Tpex and Tex‐term cells in subsequent experiments by antibody labeling PD‐1 and Tim‐3.

Flow cytometry analysis revealed that CK2B was strongly expressed in PD‐1^+^Tim‐3^+^ Tex‐term cells (*P* < 0.05) in a CD8^+^ T‐cell exhaustion model (Figure 2B). Subsequent treatment of CD8^+^ T cells induced into exhaustion with CX4945 (a CK2α phosphorylation inhibitor) and siRNA revealed a decrease in the percentage of PD‐1^+^Tim‐3^+^ Tex‐term cells among treated CD8^+^ T cells. Conversely, no significant differences were presented in the subpopulation of CD8^+^ T cells under the uninduced exhaustion model (Figure 2C,D). This result also suggests that the application of CX4945 and siRNA helps prevent the exhaustion of CD8^+^ T cells but does not have a significant effect on normally activated CD8^+^ T cells.

To further elucidate whether this subpopulation change in CD8^+^ T cells was associated with apoptosis and proliferation, we performed corresponding experiments. We first induced the transformation of CD8^+^ T cells into Tpex cells in vitro using a tumor‐conditioned medium and found that siCK2B contributed to delay the differentiation process of CD8^+^ T‐cell to Tpex cells (Figure 2E). Next, we observed the effect of siCK2B on apoptosis of CD8^+^ T cells, and the results showed that knockdown of CK2B had no significant effect on the apoptosis of CD8^+^ T cells (Figure 2F). Similarly, we examined the expression of Ki67 in each subpopulation and found no significant differences among the CK2B knockdown and control groups (Figure 2G). Furthermore, we found that after restimulation of Tpex cells by αCD3/CD28, CD8^+^ T cell expansion folds were significantly increased in the knockdown of CK2B expression group compared to the control group (Figure 2H,I). A similar trend was observed in Tex‐term cells, although the augmentation was not as dramatic as in Tpex cells (Figure 2H,I). This result suggests that knockdown of CK2B expression helps CD8^+^ Tex cells to enhance their sensitivity to external stimuli and proliferative potential. Together, these results suggest that knockdown of CK2B expression prevents the differentiation of CD8^+^ T cells into Tex cells rather than increasing apoptosis or enhancing the proliferative capacity of other cell populations.

### 2.3 Inhibition of CK2 or Knockdown of CK2B Expression Prevents or Even Reverses Terminal Exhaustion of CD8^+^ T Cells and Enhances Response to Anti‐PD‐1 Therapy

**Figure 3:**
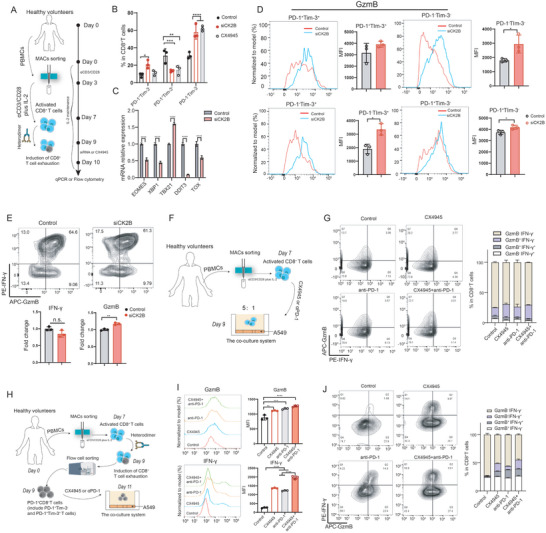
CK2B expression inhibition enhances effector function of CD8^+^ T cells in vitro. A) Schematic diagram of the experimental design of CD8^+^ T cells after induction of exhaustion by siRNA and CX4945 treatment. B) Flow cytometry analysis of the effects of siRNA and CK2 inhibitors on CD8^+^ Tex cells after induced exhaustion. C) mRNA expression levels of multiple genes in CD8^+^ Tex cells examined through RT‐qPCR after treatment with siRNA for 24 h. D) Flow cytometry analysis of GzmB expression levels in different subpopulations of CD8^+^ T cells after siRNA treatment. E) Flow cytometry analysis of the effect of CK2B expression knockdown on IFN‐γ and GzmB in normal culture of activated CD8^+^ T cells. F) Schematic diagram of the experimental design for co‐culture of activated CD8^+^ T cells with A549 cells. G) Flow cytometry analysis of the effects of CK2 inhibitors and anti‐PD‐1 mAbs (20 µg mL^−1^) on the function of CD8^+^ T cells in a co‐culture model of normally cultured CD8^+^ T cells with A549 cells. H) The schematic diagram of the experimental design for co‐culture of PD‐1^+^CD8^+^ T cells treated with CX4945 or anti‐PD‐1 mAbs with A549 cells. I,J) Flow cytometry analysis of functional changes in CD8^+^ T cells after addition of CK2 inhibitors and anti‐PD‐1 mAbs in a co‐culture model of CD8^+^ Tex cells with A549. CX4945: CK2 inhibitor; MFI: mean fluorescence intensity. *P < 0.05, **P < 0.01, ***P < 0.001, and ****P < 0.0001 (Student's t test); n.s., not significant (*P* > 0.05).

In the above experiments, we found that the application of CK2 inhibitors or knockdown of CK2B expression during the induction of CD8^+^ T‐cell exhaustion helped prevent the exhaustion of CD8^+^ T cells. Furthermore, we investigated whether the inhibition of CK2B expression in CD8^+^ Tex cells could reverse the exhaustion of CD8^+^ T cells. We treated in vitro‐induced exhausted CD8^+^ T cells with siRNA and CX4945, resulting in a marked decrease in the proportion of Tex‐term cells and a significant increase in the proportion of PD‐1^−^Tim‐3^−^ double‐negative cells (**Figure** **3**A,B). We also analyzed the expression levels of mRNAs associated with T‐cell exhaustion in siRNA‐treated CD8^+^ Tex cells, and found that the expression levels of *TOX*, *EOMES*, *XBP1*, and *DDIT3* were significantly reduced, whereas that of *TBX21* significantly increased (Figure 3C). Furthermore, following siRNA treatment, the expression of GzmB significantly increased in all subpopulations except Tex‐term cells (Figure 3D). The above results suggest that knockdown of CK2B expression or inhibition of CK2 prevents or even reverses terminal exhasution of CD8^+^ T cells and up‐regulates the expression of T cell‐activated transcription factor TBX21 and cytokine GzmB.

To further clarify the target cells (Tpex or Tex‐term cells) regulated by CK2B, we sorted CD8^+^ T cells by flow cytometry after inducing T cell exhaustion in vitro and treated the sorted Tpex and Tex‐term cells with CX4945 (Figure S4B, Supporting Information). We found that the proportion of Tex‐term cells decreased and the proportion of Tpex cells and double‐negative T cells increased among the sorted PD‐1^+^Tim‐3^+^ T cells after treatment with CX4945 (Figure S4C, Supporting Information). Moreover, similar results were observed in the sorted PD‐1^+^Tim‐3^−^ T cells, where the percentage of Tpex cells and double‐negative T cells increased, while the proportion of Tex‐term cells decreased (Figure S4C, Supporting Information). This result indicates that CK2 inhibitors inhibit or even reverse the differentiation process of double‐negative T cells and Tpex cells to Tex‐term cells. To further validate our in vitro results, we designed and performed an animal experiment in which Tpex and Tex‐term cells were sorted by flow cytometry and adoptive into the CD45.1 mice bearing LLC‐OVA transplanted tumors (Figure S4D, Supporting Information). The results showed that the Tpex plus CX4945 group exhibited the best anti‐tumor effect compared to all other groups (EC 75%) (Figure S4E,F, Supporting Information). Furthermore, there was no significant difference in efficacy between the Tex‐term plus CX4945 group and the Tex‐term group, although a trend toward reduced tumor growth volume was observed (Figure S4E,F, Supporting Information).

Next, we performed further analysis of the tumor‐infiltrating immune cells. Compared to the Tpex‐only adoptive cellular therapy group, the proportion of CD45.2^+^CD8^+^ T cells infiltrating the tumor was significantly higher in the Tpex plus CX4945 group (Figure S4G,H, Supporting Information). Meanwhile, the proportion of Tex‐term cells among the adoptively transferred CD8^+^ T cells was significantly reduced, while the proportion of Tpex cells increased, and there was a trend toward an increase in double‐negative T cells (*P *= 0.09) (Figure S4I, Supporting Information). Additionally, we observed a trend toward an increase in the proportion of CD45.2^+^CD8^+^ T cells infiltrating the tumor in the Tex‐term plus CX4945 group compared to the Tex‐term‐only adoptive cellular therapy group (though not statistically significant) (Figure S4G,H, Supporting Information). At the same time, we observed that compared to the Tex‐term‐only adoptive cellular therapy group, the Tex‐term plus CX4945 group had a significantly lower proportion of CD8^+^ Tex‐term cells, a significant increase in double‐negative T cells, and a trend toward an increased proportion of Tpex cells (although this trend did not reach statistical significance) (Figure S4H,I, Supporting Information). These results help explain why the Tpex plus CX4945 group exhibited the best therapeutic effect, which was closely associated with the increased proportion of tumor‐infiltrating CD45.2^+^CD8^+^ T cells. Moreover, these findings further demonstrate that CK2 inhibitors can help prolong, and even partially reverse, the differentiation process of CD8^+^ T cells into Tex‐term cells, thereby enhancing the efficacy of adoptive therapy. Together, these findings suggest that CK2 inhibitors or CK2B knockdown prevent the process of T‐cell exhaustion or even promote the differentiation of Tex‐term cells into Tpex or double‐negative T cells.

Next, we knocked down CK2B expression during normal culture of CD8^+^ T cells and found no significant effect on the expression of IFN‐γ but a slight raised in GzmB expression (Figure 3E). Moreover, when CD8^+^ T cells cultured under normal conditions were co‐cultured with tumor cells, there was no significant increase in CD8^+^ T cell function in the CX4945 group, the anti‐PD‐1 therapy group, or the CX4945 plus anti‐PD‐1 therapy group compared to the control group. (Figure 3F,G). These results suggest that the knockdown of CK2B expression or application of CK2 inhibitors in normal cultured CD8^+^ T cells did not have a marked effect on the overall function of CD8^+^ T cells or the response to anti‐PD‐1 therapy.

Finally, we co‐cultured CD8^+^PD‐1^+^ T cells and A549 cells after induced exhaustion in vitro and treated them with CX4945 and anti‐PD‐1 mAbs. We found that the expression of IFN‐γ and GzmB significantly increased in CD8^+^ T cells from the CX4945 group, the anti‐PD‐1 therapy group, and the CX4945 plus anti‐PD‐1 therapy groups compared to the control group (Figure 3H,I). Notably, CD8^+^ T cells in the CX4945 plus anti‐PD‐1 therapy group exhibited the highest levels of IFN‐γ and GzmB among all the treatment group (Figure 3J). These results suggest that the application of CK2 inhibitors helps restore the function of CD8^+^ T cells in an exhausted state and further enhances their response to anti‐PD‐1 therapy.

### 2.4 CK2 Inhibitor or CK2B Knockdown Limits CD8^+^ T Cell Exhaustion and Improves the Efficacy of Anti‐PD‐1 Therapy in NSCLC In Vivo

Based on the aforementioned in vitro experimental results, we established a mouse model with LLC‐transplanted tumors to corroborate the observed phenomena and mechanisms from the in vitro experiments. Furthermore, we further explore the anti‐tumor effects of CK2 inhibitors plus anti‐PD‐1 therapy.

We found that the tumor volume significantly reduced (effective tumor control rate of 83.3%, 5/6) in mice from the CX4945 plus anti‐PD‐1 therapy group; in addition, the weight of the tumor exhibited substantial decrease compared to the control group (*P* < 0.05), whereas there was no considerable difference in the body weight of the mice (Figure S5A–D, Supporting Information). These results indicated that the combination treatment significantly inhibited tumor progression without significant toxic side effects.

In addition, flow cytometry analysis showed a considerable increase in the percentage of tumor‐infiltrating CD3^+^ and CD8^+^ T cells in the combination group compared to the other three groups (Figure S5E, Supporting Information). Meanwhile, the percentage of IFN‐γ^+^GzmB^+^CD8^+^ T cells within tumor tissues was increased, whereas that of PD‐1^+^Tim‐3^+^CD8^+^ T cells was decreased in the CX4945 group and the CX4945 plus anti‐PD‐1 therapy group compared to the control group (Figure S5F, Supporting Information). Furthermore, we also found in the TC‐1 transplantation tumor mouse model, where the percentage of CD8^+^PD‐1^+^Tim‐3^+^ T cells infiltrated within tumor tissues significantly decreased after receiving CX4945, whereas that of IFN‐γ^+^GzmB^+^ T cells significantly raised (Figure S6A, Supporting Information). These results indicated that CK2 inhibitors can effectively improve the exhaustion of CD8^+^ T cells, enhance the function of CD8^+^ T cells, and significantly enhance the efficacy of anti‐PD‐1 therapy in NSCLC.

**Figure 4:**
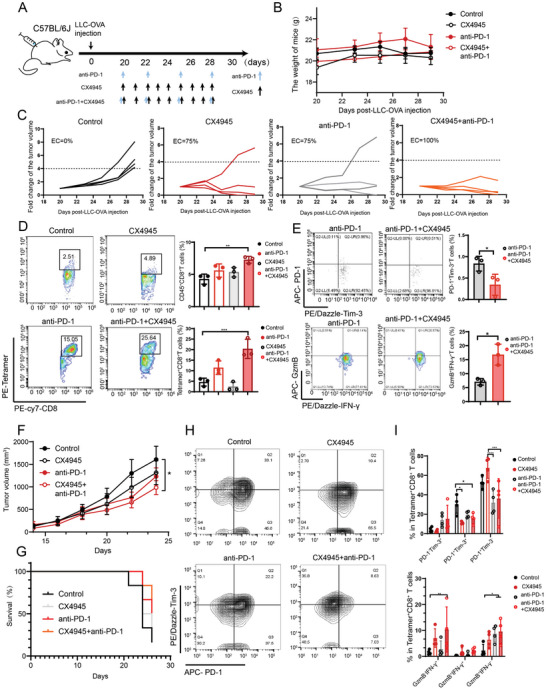
Effect of CK2 inhibitors on antigen‐specific CD8^+^ T‐cell exhaustion and efficacy of anti‐PD‐1 therapy in animal experiments. C57BL/6J mice were inoculated with LLC‐OVA tumor cells. On day 20, the mice were administered with CX4945, anti‐PD‐1 mAbs, or PBS control. On day 29, mice were sacrificed for flow cytometry analyses of TILs. A) Scheme representing the mouse LLC‐OVA transplantation tumor model construction and drug administration pattern. B) Body weight–time change curve in mice. C) Individual change curve of tumor volume in mice (fold change of the tumor volume not exceeding 4 was defined as the tumors were effectively controlled to be recorded). D) Representative flow cytometry graphs showing the frequencies of CD3^+^CD8^+^ T and CD3^+^tetramer^+^CD8^+^ T cells infiltrated within the tumors (left) and statistical plots (right). E) Flow cytometry analysis of the expression levels of IFN‐γ and GzmB, as well as PD‐1 and Tim‐3, in tetramer^+^CD8^+^ T cells among different treatment groups (*n* = 3). F) Tumor volume–time change curve in mice with TC‐1‐OVA tumor cells (Data are expressed as mean ± s.e.m.). G) Survival curves in mice with TC‐1‐OVA transplanted tumor cells. Typical flow cytometry graphs of PD‐1 and Tim‐3 H), and statistical charts of the expression levels of IFN‐γ and GzmB, as well as PD‐1 and Tim‐3, in tetramer^+^CD8^+^ T cells from different treatment groups (I, *n* = 4 independent animals). EC: effective tumor control rate. *P < 0.05, **P < 0.01, ***P < 0.001, and ****P < 0.0001 (one‐way ANOVA or Student's t test).

To further investigate the effects of CK2 inhibitors on tumor‐infiltrating antigen‐specific CD8^+^ T cells, we constructed an LLC‐OVA‐transplanted tumor mouse model. The CX4945 plus anti‐PD‐1 therapy had a favorable anti‐tumor effect in the LLC‐OVA transplantation tumor mouse model (EC 100%), and no significant adverse effects (**Figure** **4**A–C). We found that the combination treatment group had a higher percentage of tumor‐infiltrating CD8^+^ T cells and tumor‐infiltrating tetramer^+^CD8^+^ T cells (tumor‐infiltrating antigen‐specific CD8^+^ T cells) than the control group (Figure 4D). In addition, the combination treatment group had a lower proportion of tumor‐infiltrating CD8^+^ Tex cells and higher proportion of IFN‐γ^+^GzmB^+^ T cells in the tumor site than the anti‐PD‐1 therapy group (Figure 4E). We also performed a corresponding validation in a TC‐1‐OVA transplantation tumor mouse model and observed a similar phenomenon. The combination therapy group significantly inhibited tumor progression, reduced mortality in mice, decreased the proportion of tetramer^+^CD8^+^ Tex‐term cells, and increased the proportion of tetramer^+^IFN‐γ^−^GzmB^+^ T and tetramer^+^IFN‐γ^+^GzmB^−^ T cells (Figure 4F–I). These results suggested that CX4945 inhibited the exhaustion of tumor antigen‐specific CD8^+^ T cells and enhanced the efficacy of anti‐PD‐1 therapy.

To further confirm whether the effector of anti‐tumor by combination therapy was CD8^+^ T‐cell dependent, we used a CD8^+^ T cells depletion antibody to remove CD8^+^ T‐cells in mice. We found that the anti‐tumor effect of the combination therapy group was significantly inhibited by the removal of the CD8^+^ T‐cells (*P *< 0.01), both in the LLC‐OVA cell line and in the TC‐1‐OVA cell line (Figure S6B,C, Supporting Information). This result suggests that the anti‐tumor efficacy of CX4945 combined with anti‐PD‐1 therapy is dependent on CD8^+^ T cells in mice. In addition, in our previous experiments, we have observed that the TC‐1‐OVA cell line is more resistant to anti‐PD‐1 therapy. Therefore, to evaluate whether CX4945 could enhance the efficacy of anti‐PD‐1 therapy in tumors resistant to it, we administered CX4945 in a TC‐1‐OVA transplantation tumor model in mice with tumors that continued to grow rapidly despite two times of anti‐PD‐1 therapy. Interestingly, we found that tumors resistant to anti‐PD‐1 therapy were effectively controlled after treatment with CX4945 (Figure S6D, Supporting Information). This result also suggests again that the application of CX4945 will enhance the efficacy of anti‐PD‐1 therapy in mice burdened with tumors that are resistant to and have low responsiveness to anti‐PD‐1 therapy. To further determine whether the administration sequence affects the efficacy of CX4945 combined with anti‐PD‐1 therapy, we utilized a TC‐1–OVA‐transplanted tumor mouse model known for its low response rate to anti‐PD‐1 therapy. Our results demonstrated that administering CX4945 either before or after anti‐PD‐1 therapy effectively inhibited tumor progression (with no significant differences among the three groups), alleviated CD8^+^ T cell exhaustion, and enhanced CD8^+^ T cell effector functions (Figure S7A–E, Supporting Information). These findings preliminarily suggest that the anti‐tumor effects of CX4945 plus anti‐PD‐1 therapy are not strongly dependent on the sequence of administration.

Additionally, previous study has reported that CX4945 can improve the TME by downregulating PD‐L1 expression on tumor cells.^[^ ^22^ ^]^ To further investigate whether CX4945's regulation of CD8^+^ T cell exhaustion in vivo depends on the reduction of PD‐L1 expression on tumor cells, we established an LLC‐OVA‐transplanted tumor mouse model and restored PD‐L1 expression in tumor tissues by administering chloroquine to counteract the downregulation caused by CX4945. Interestingly, even after PD‐L1 expression was restored, CX4945 continued to alleviate CD8^+^ T cell exhaustion and enhance their effector functions (Figure S7F–H, Supporting Information). These results further demonstrate that the regulatory effect of CK2 inhibitors on CD8^+^ T cell exhaustion is independent of PD‐L1 expression on tumor cells.

**Figure 5:**
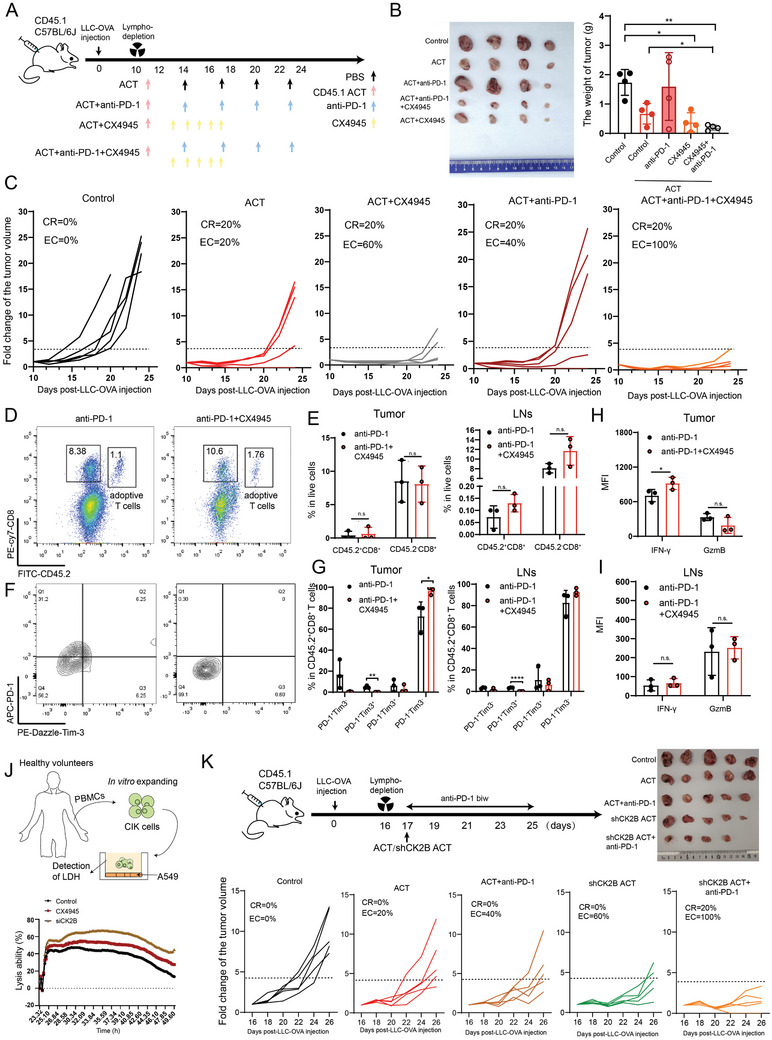
Effect of CK2B knockdown in CD8^+^ T cells on efficacy of adoptive antigen‐specific CD8^+^ T‐cell therapy. On day 0, CD45.1^+^C57BL/6J mice were inoculated with LLC‐OVA tumor cells. On day 10, the mice were sublethally lymphodepleted and received adoptive CD45.2^+^ activated OT‐I CD8^+^ T cells followed by administration of CX4945, anti‐PD‐1 mAbs or PBS control. On day 24, mice were sacrificed for flow cytometry analyses of TILs. A) Schematic description of mouse LLC‐OVA transplantation tumor model construction and drug administration pattern. B) Image of the collected mouse tumors (left) and a statistical graph of the weight of the tumors (right). C) Individual change curve of tumor volume in mice (fold change of the tumor volume not exceeding 4 was defined as tumors that were effectively controlled to be recorded). D,E) Representative flow cytometry graphs showing the frequencies of adoptive CD45.2^+^CD8^+^ OT‐1 cells infiltrated within the tumor sites or LNs among anti‐PD‐1 therapy and anti‐PD‐1 therapy plus CX4945 groups. F,G) Flow cytometry analysis of adoptive OT‐1 CD45.2^+^CD8^+^ cell exhaustion in tumor sites or LNs from anti‐PD‐1 therapy group and anti‐PD‐1 therapy plus CX4945 group. H,I) Statistical charts of the expression levels (MFI) of IFN‐γ and GzmB in CD45.2^+^CD8^+^ T cells in tumor sites or LNs from anti‐PD‐1 therapy group and anti‐PD‐1 therapy plus CX4945 group. J) PBMCs from healthy volunteers were stimulated and expanded in vitro to obtain CIK cells, which were co‐cultured with A549 cells at a 40: 1 ratio (upper); the cytotoxicity of the CIK cells, treated with either siRNA or CX4945, was assessed by measuring lactate dehydrogenase (LDH) release in the co‐culture supernatants (bottom). K) Subcutaneous growth of tumor cells (LLC‐OVA) in each group of CD45.1 mice received adoptive activated OT1 CD45.2^+^CD8^+^ T cells (*n* = 5), OT1 CD45.2^+^CD8^+^ T cells plus anti‐PD‐1 mAbs (*n* = 5), OT‐1 shCK2B CD45.2^+^CD8^+^ T cells (*n* = 5) or adoptive activated OT‐1 shCK2B CD45.2^+^CD8^+^ T cells plus anti‐PD‐1 mAbs (*n* = 5). LNs: lymph nodes; ACT: adoptive cellular therapy; EC: effective tumor control rate; CR: complete tumor regression. *P < 0.05, **P < 0.01, ***P < 0.001, and ****P < 0.0001 (one‐way ANOVA or Student's t test).

To further explore the effects of CK2 inhibitors and shCK2B on adoptive antigen‐specific CD8^+^ T cells, we constructed an animal model, as shown in **Figure** **5**A. We found that CX4945 plus anti‐PD‐1 therapy significantly improved the inhibition of tumor growth by adoptive OT‐1 CD8^+^ effector T cells (CR 20% and EC 100%) (Figure 5B,C). In a subsequent analysis of the immune cell situation in tumor tissues or draining lymph nodes (LNs), we found that CX4945 plus anti‐PD‐1 therapy significantly reduced the proportion of CD8^+^ Tex‐term cells but did not significantly promote adoptive CD8^+^ T‐cell infiltration compared with anti‐PD‐1 monotherapy, although there was a tendency for increased infiltration (Figure 5D–G). In addition, we observed that CX4945 plus anti‐PD‐1 therapy significantly enhanced the expression of IFN‐γ in adoptive CD8^+^ T cells in tumor tissues compared with anti‐PD‐1 monotherapy (Figure 5H,I).

Furthermore, we found that the proportion of Tpex cells and Tex‐term cells of CD45.1^+^CD8^+^ T cells was significantly lower in tumor tissues treated with anti‐PD‐1 mAbs or CX4945 compared to controls, whereas the combination treatment group had a higher expression of IFN‐γ (Figure S8A, Supporting Information). We also found a lower proportion of CD45.1^+^CD8^+^ Tex‐term cells, and a trend toward a decreased proportion of Tpex cells in the draining LNs in the CX4945 plus anti‐PD‐1 therapy group compared with anti‐PD‐1 monotherapy (Figure S8B, Supporting Information). Due to the embryonic lethality associated with CK2B knockout mice and the impact of CK2B deficiency on T cell differentiation,^[^ ^23^ ^]^ we chose to perform lentiviral knockdown of CK2B to observe the exhaustion of adoptive CD8^+^ T cells. We found that the percentage of Tex‐term cells in adoptive CD8^+^ T cells in tumor tissues was significantly lower after knockdown of CK2B compared with the control group (*P *< 0.05) (Figure S8C, Supporting Information). Taken together, these results suggest that the administration of CK2B knockdown or CK2 inhibitors contribute to delaying the adoptive CD8^+^ T‐cell exhaustion and enhances the efficacy of anti‐PD‐1 therapy in both draining LNs and tumor tissues.

In addition, based on previous literature,^[^ ^24^, ^25^ ^]^ we cultured non‐MHC‐restricted killer cell‐cytokine‐induced killer (CIK) cells to investigate the effect of inhibiting CK2 or knocking down CK2B expression on the killing effect of CIK cells. We found that CK2B knockdown significantly enhanced the killing effect of CIK cells on tumor cells, which was even stronger than the enhancement of CIK cell killing by CX4945 (Figure 5J). We observed a similar phenomenon, that is, the knockdown of CK2B by adoptive CD8^+^ T cells significantly enhanced the efficacy of anti‐PD‐1 therapy (CR 20% vs 0%, EC 100% vs 40%) (Figure 5K). These results also suggest that the administration of CK2B knockdown or CK2 inhibitors has good anti‐tumor efficacy in adoptive cell therapy.

Our research team earlier performed a single‐center, open‐label, phase 1 B clinical trial exploring CIK cell therapy paired with anti‐PD‐1 mAbs plus chemotherapy for patients with advanced untreated NSCLC and obtained favorable results.^[^ ^26^ ^]^ We retrospectively collected peripheral blood mononuclear cells (PBMCs) from 10 patients, compiled relevant clinical data (Table S1, Supporting Information), examined the expression of CK2B in CD8^+^ T cells, separated them into groups with high and low expression based on the median CK2B expression, and performed survival analysis (Figure S9A, Supporting Information). The high‐expression group showed a trend toward (*P* = 0.154) poorer PFS than the low‐expression group, although the difference was not significant (Figure S9B, Supporting Information).

We further categorized patients into responder [includes partial response (PR) or complete response (CR)] and non‐responder [including stable disease (SD) or progression disease (PD)] groups based on their best treatment response. Comparing the expression of CK2B before and after treatment, we found that CK2B expression in peripheral blood CD8^+^ T cells from non‐responder group was significantly raised than responder group before treatment (Figure S9C, Supporting Information).

Additionally, we examined CK2B expression levels in CD8^+^ T cells and the percentages of CD3^+^CD8^+^ T cells, PD‐1^+^Tim‐3^−^ T cells, PD‐1^−^Tim‐3^+^ T cells, TCF‐1^+^PD‐1^−^ T cells, and TCF‐1^−^PD‐1^+^ T cells in both groups before and after treatment. In the responder group, CK2B expression in peripheral blood CD8^+^ T cells increased after treatment, along with the percentage of CD8^+^ T cells. In contrast, there was no significant change in either the percentage of CD8^+^ T cells or CK2B expression in the non‐responder group (Figure S9D, Supporting Information).

Moreover, although the percentages of PD‐1^+^Tim‐3^−^ T cells, PD‐1^−^Tim‐3^+^ T cells, TCF‐1^+^PD‐1^−^ T cells, and TCF‐1^−^PD‐1^+^ T cells showed no significant difference after treatment compared to before treatment, we observed a trend where these percentages tended to decrease in the non‐responder group and increase in the responder group (Figure S9D, Supporting Information). These results suggest that the upregulation of CK2B expression in peripheral blood after treatment indicates a favorable immunotherapeutic effect, which may be closely related to the expansion of Tpex cells.

Taken together, based on the above results, high expression of CK2B predicted a poor prognosis, whereas an increased number of Tpex cells accompanied by a certain degree of increased CK2B expression after treatment predicted improved immunotherapeutic efficacy.

### 2.5 HDAC8 is Involved in the Regulation of CD8^+^ T‐Cell Exhaustion by CK2B

**Figure 6:**
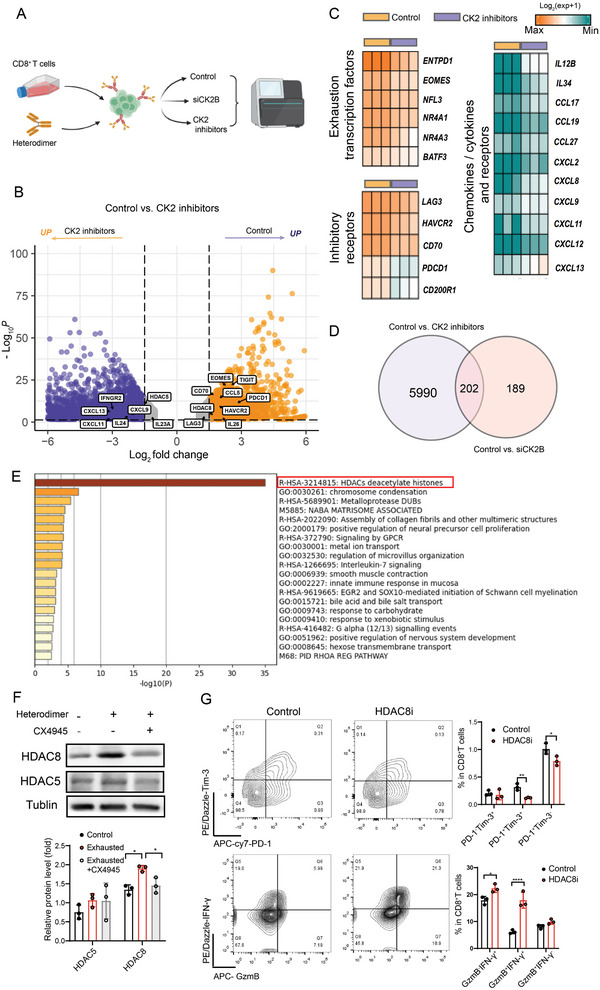
HDAC8 is involved in the regulation of CD8^+^ T‐cell exhaustion by CK2B. A) Schematic experimental setup of RNA‐seq after treatment with CK2 inhibitors or siRNA in CD8^+^ Tex cells. B) Volcano plots showing the fold change of genes described in the hierarchical cluster analysis on the corrected *P* values. Blue dots represent genes significantly up‐regulated in the CK2 inhibitor group, and yellow dots indicate genes significantly up‐regulated in the control group (corrected *P*‐value < 0.05, FC > 1.5). C) Heatmap showing transcript expression of the indicated genes, and transcripts of all the indicated genes were calculated to be significantly different by edgeR (corrected *P* < 0.05). D) Venn diagrams showing (control group vs CK2 inhibitor group and control group vs siCK2B group) the overlap of differential genes between two groups. E) Pathway enrichment analysis of differential genes using the online Metascape tool. F) Western blot analysis of the expression levels of HDAC5, HDAC8, and Tublin in CD8^+^ T cells were treated with CX4945 (10 µM, at time 24 h) and heterodimer (1 µg mL^−1^, at time 0); cellular proteins were collected at 48 h. G) Flow cytometry analysis of exhaustion and cytokine production in CD8^+^ T cells in the exhaustion model after treatment with HDAC8‐specific inhibitor (PCI‐34051, 20 nm) for 24 h. *P < 0.05, **P < 0.01, ***P < 0.001, and ****P < 0.0001 (Student's t test).

A CD8^+^ T‐cell exhaustion model was established in vitro, and RNA‐seq was performed on CD8^+^ Tex cells treated with CK2 inhibitors or siRNA (**Figure** **6**A). We found that genes encoding immunosuppressive receptors (e.g., *PDCD1*, *TIGIT*, *HAVCR2*, and *CD70*) and those related to T‐cell exhaustion (e.g., *EOMES*, *BATF3*, and *NFL3*) were downregulated in the CX4945 group by differential gene enrichment analysis of the RNA‐seq data, whereas genes encoding chemokines and cytokines (e.g., *CXCL9*, *CXCL11*, and *CXCL12*) were upregulated (Figure 6B,C). This result further suggests that the inhibition of CK2 helps prevent or even reverse CD8^+^ T‐cell exhaustion and improve CD8^+^ T‐cell function.

To further confirm the downstream signaling pathways and target genes regulated by CK2B, we examined the intersection of the two sets of differential gene datasets and found that the number of differential genes common to both datasets was 202 (Figure 6D). Pathway enrichment analysis of the differentially expressed genes showed that they were mainly enriched in histone deacetylase (HDAC)‐related pathways (Figure 6E). CK2 regulates the phosphorylation and expression levels of HDAC1/2, especially under hypoxic conditions, HDAC1 and HDAC2 activation is dependent on CK2 phosphorylation.^[^ ^27^, ^28^ ^]^ In addition, HDAC3 activation is regulated by CK2, which has a protein kinase CK2 phosphorylation receptor site at the Ser424 site of the HDAC3 protein.^[^ ^29^ ^]^

We also analyzed the correlation between *CSNK2B* expression and HDAC family‐related genes in the TIMER2.0 public database of multi‐cancer, and we found that the expression of *CSNK2B* showed a positive correlation with *HDAC1*, *HADC2*, *HDAC5*, *HDAC6*, and *HDAC8* in LUAD and LUSC (Figure S10A,B, Supporting Information). In view of this, coupled with the differential gene enrichment results from our RNA‐seq data, we directed our focus toward HDAC5 and HDAC8, two genes downstream of CK2. Next, we confirmed by western blot experiments that the addition of a CK2 inhibitor significantly downregulated HDAC8 expression in the exhaustion model (given the positive feedback loop between CK2B expression and T cell exhaustion, this model can also be viewed as a method to induce elevated CK2B expression), but it had no significant effect on HDAC5 expression (Figure 6F). In addition, we used siRNA to knock down CK2B expression in vitro, detected it using flow cytometry, and found that CK2B knockdown inhibited HDAC8 expression and increased TBX21 expression (Figure S11A, Supporting Information).

Next, we added the HDAC8‐specific inhibitor PCI‐34051 in an induced CD8^+^ T‐cell exhaustion model. We found a reduction in the percentage of CD8^+^ Tex cells, and an increase in the production of cytokines IFN‐γ and GzmB (Figure 6G). Similarly, after knocking down the expression of HDAC8 using siRNA, we found that the percentage of CD8^+^ Tex cells decreased, along with increased production of IFN‐γ and GzmB (Figure S11B,C, Supporting Information). We also observed that HDAC8 expression was positively correlated with *CSNK2A1*, *CSNK2A2*, and *CSNK2B* in multiple cancer types in public databases (Figure S12A, Supporting Information). On the contrary, it was negatively correlated with cytokine‐encoding related genes such as *IFNG, GZMB*, *PRF1*, and *TNF* in most tumor types. In addition, HDAC8 expression was positively correlated with the genes encoding T‐cell exhaustion‐related genes, including *TOX* and *DDIT3*, and negatively correlated with the transcript genes encoding T‐cell activation‐related genes, such as *TBX21*, in multiple tumor types (Figure S12B,C, Supporting Information). These findings also suggest that HDAC8 is a crucial regulator in CK2B‐mediated CD8^+^ T‐cell exhaustion.

**Figure 7:**
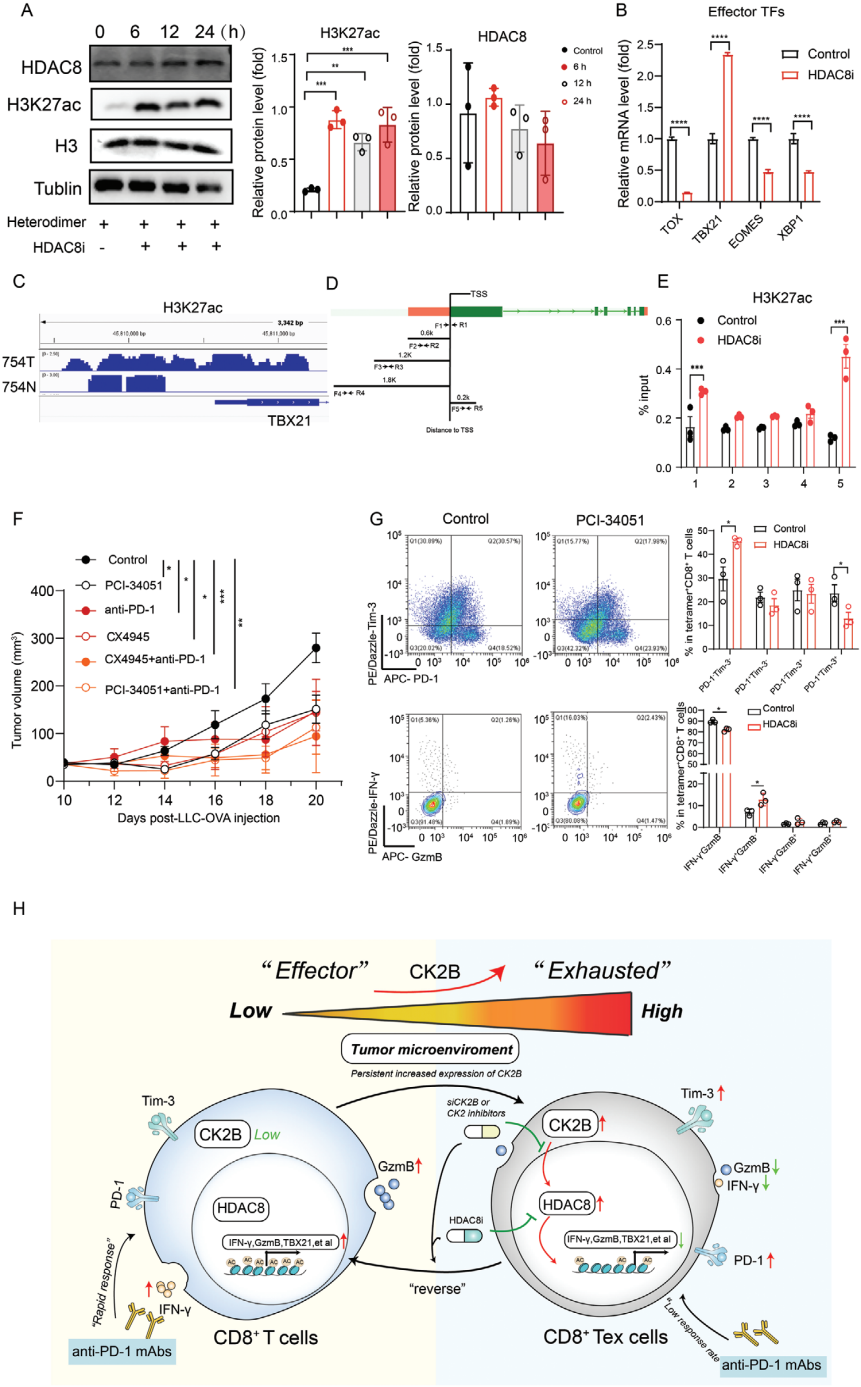
TBX21 expression is regulated by HDAC8‐mediated histone acetylation. A) Western blot analysis of the protein expression levels of HDAC8, H3K27ac, H3, and Tublin in CD8^+^ T cells treated with PCI‐34051 (20 nm); cellular proteins were collected at indicated time points (0–24 h). B) mRNA expression levels of effector TF genes in CD8^+^ T cells examined through RT‐qPCR after treatment with PCI‐34051 for 24 h. C) Overlapping ChIP sequencing peaks for H3K27ac on TBX21. D) Design of paired primers at H3K27ac binding sites within TBX21 promoter. E) HDAC8 inhibition upregulated H3K27ac binding to TBX21 promoter, as determined by ChIP‐qPCR. F) Subcutaneous growth of tumor cells (LLC‐OVA) in each group of mice treated with PCI‐34051 (200 µg/per mouse, *n* = 5), anti‐PD‐1 mAbs (150 µg/per mouse, *n* = 5), CX4945 (10 mg kg^−1^, *n* = 5), CX4945 plus anti‐PD‐1 mAbs (*n* = 5), and PCI‐34051 plus anti‐PD‐1 mAbs (*n* = 5). G) Flow cytometry analysis of the expression levels of IFN‐γ and GzmB, as well as PD‐1 and Tim‐3, in tetramer^+^CD8^+^ T cells from each treatment groups. H) In the tumor microenvironment, CD8^+^ effector T cells, under the influence of multiple external physicochemical factors, gradually increase CK2B expression, which inhibits TBX21 expression by regulating HDAC‐mediated epigenetic reprogramming, thereby promoting T cell exhaustion and limiting the efficacy of anti‐PD‐1 therapy. HDAC8i: HDAC8 inhibitors; TF: transcription factor. *P < 0.05, **P < 0.01, ***P < 0.001, and ****P < 0.0001 (one‐way ANOVA or Student's t test).

HDAC8 inhibitors can remodel the epigenetic program of tumor cells and effectively restore H3K27 acetylation, thereby promoting the infiltration of CD8^+^ T cells into tumor sites, which, in combination with anti‐PD‐1 therapy, induces an effective and long‐lasting anti‐tumor response.^[^ ^30^ ^]^ Therefore, we explored the relationship between HDAC8 and H3K27ac in CD8^+^ T cells. We observed that HDAC8 expression was not marked difference in CD8^+^ Tex cells treated with an HDAC8 inhibitor (PCI‐34051), whereas the H3K27ac protein level significantly increased (**Figure** **7**A). Furthermore, we analyzed the transcription factors (TF) expression using RT‐qPCR and found a significant downregulation of transcripts associated with T cell exhaustion and in contrast a marked upregulation of TBX21 expression (Figure 7B). After siRNA knockdown of HDAC8 expression, we observed a similar phenomenon, that is, the knockdown of HDAC8 significantly upregulated the H3K27ac and TBX21 expression (Figure S13A, Supporting Information).

Furthermore, we predicted the region where H3K27ac binds to TBX21 using data from the public database GSM50096 and designed the relevant ChIP‐qPCR primers (Figure 7C,D). Through ChIP‐qPCR experiments, we found that HDAC8 inhibition significantly enhanced the enrichment of H3K27ac in the TBX21 promoter region, which in turn activated TBX21 mRNA expression (Figure 7E). Finally, we performed related experiments in the animals and found that HDAC8 inhibitors combined with anti‐PD‐1 therapy markedly inhibited tumor progression (no significant difference in efficacy from the CX4945 plus anti‐PD‐1 therapy group), and application of PC1‐34051 effectively reducing the proportion of CD8^+^ Tex cells infiltrated within the tumor, as well as enhancing the production of IFN‐γ (Figure 7F,G). In addition, we found that compared to the anti‐PD‐1 monotherapy group, both the PC1‐34051 plus anti‐PD‐1 therapy group and the CX4945 plus anti‐PD‐1 group significantly promoted the infiltration of antigen‐specific CD8^+^ T cells, as well as decreasing the percentage of Tpex and Tex‐term in CD8^+^ T cells, while promoting IFN‐γ production (Figure S13B–D, Supporting Information). These results suggest that HDAC8 is involved in CK2‐mediated CD8^+^ T‐cell exhaustion by inhibiting the transcription of TBX21, whereas application of HDAC8 inhibitors significantly suppressed CD8^+^ T‐cell exhaustion and enhanced the efficacy of anti‐PD‐1 therapy.

## 3 Discussion

Lung cancer is among the most widespread malignant tumors worldwide, with NSCLC comprising about 85% of all cases. Unfortunately, most patients with NSCLC are already at an advanced stage, when they are primarily diagnosed. Despite significant advancements in immunotherapy, targeted therapy, and radiotherapy, the prognosis at 5 years in patients with advanced NSCLC remains below 6%.^[^ ^2^, ^31^, ^32^ ^]^ Improving treatment outcomes for NSCLC and increasing patient survival rates are critical clinical challenges.

In clinical practice, the efficacy of anti‐PD‐1 therapies has significantly underperformed compared to researchers' expectations. Most patients who initially respond to ICIs ultimately develop resistance, with only a few (only 10% to 30%) benefiting from this therapy in the long term.^[^ ^3^ ^]^

Researchers have performed in‐depth explorations to elucidate the mechanisms of resistance to ICIs, and T‐cell exhaustion is considered one of the crucial factors limiting the efficacy of ICIs. In particular, during anti‐PD‐1/PD‐L1 therapy, Tpex cells, as the main responsive cell population, undergo transient activation and large‐scale expansion, but they still differentiate into Tex‐term populations unresponsive to ICIs, which ultimately leads to resistance to ICIs.^[^ ^7^, ^8^, ^9^, ^10^ ^]^

Currently, approaches to completely overcome T cell exhaustion are lacking. Therefore, it is imperative necessity to identify new targets that can prevent or reverse T cell exhaustion. This approach could address the clinical challenges of low response rates to anti‐PD‐1 therapy and immunotherapeutic resistance.

In our study, scRNA‐seq analysis revealed that the No‐MPR group had more CD8^+^ Tex cells compared to the MPR group, while CK2B expression was significantly up‐regulated in these CD8^+^ Tex cells. Its expression increases progressively during T‐cell differentiation. Furthermore, exhaustion‐related pathways and transcripts were enriched in CK2B high‐expressing CD8^+^ T cells, suggesting a potential correlation between CK2B expression and CD8^+^ T‐cell exhaustion.

CK2B, a subunit of the CK2 protein, showed a higher level of expression than the other subunits, CK2A1 and CK2A2, in CD8^+^ T cells. Unlike CK2B, CK2A1 and CK2A2 expression was not significantly upregulated in CD8^+^ Tex cells. These results highlight the crucial role of CK2B in CD8^+^ T cell exhaustion.

Furthermore, our analysis of single‐cell data revealed a close relationship among CK2B expression in CD8^+^ T cells, oxidative phosphorylation, and ER stress. It is well established that ER stress is a critical factor in inducing T cell exhaustion within TME.^[^ ^33^, ^34^ ^]^ Based on the previous literature,^[^ ^17^ ^]^ we constructed a model to induce ER stress in CD8^+^ T cells using in vitro experiments. Our findings demonstrate that ER stress is involved in the regulation of CK2B expression in CD8^+^ T cells.

Next, we inhibited the expression of CK2B in vitro using CK2 inhibitors or siRNA, both of which contributed to a reduction in the proportion of CD8^+^ Tex cells, restoration of the effector function of CD8^+^ T cells and enhanced the efficacy of anti‐PD‐1 therapy. In particular, we found that inhibiting or knocking down CK2B expression has little effect on normally activated CD8^+^ T cells. Moreover, the CK2 inhibitors used in our study, CX4945, has been approved by the FDA for the treatment of cholangiocarcinoma and has demonstrated promising anti‐tumor activity in clinical settings.^[^ ^35^ ^]^ Therefore, our findings provide a strong experimental foundation for the combined application of CK2 inhibitors and anti‐PD‐1 therapy in clinical settings. In addition, we found that knockdown of CK2B expression improved the effector function of CIK cells in vitro experiments. Meanwhile, in a clinical trial of CIK cells combined with anti‐PD‐1 therapy performed by our team,^[^ ^26^ ^]^ by retrospectively collecting PBMCs from patients with NSCLC before and after treatment. We observed that high CK2B expression in peripheral blood CD8^+^ T cells prior to treatment predicted poor immunotherapy efficacy and prognosis, on the contrary, significant elevation of CK2B and an increase in Tpex cells after treatment predicted a better immunotherapy efficacy.

We further explored the mechanism by which CK2B regulates T‐cell exhaustion. Our RNA‐seq data revealed that CK2B regulated the downstream HDAC‐related signaling pathway and affected the expression of HDAC8 in vitro. Moreover, HDAC8 inhibitors decreased the proportion of CD8^+^ Tex cells and improved the effector function of CD8^+^ T cells.

HDAC8, the first HDAC whose 3D structure was elucidated by X‐ray crystallography, is ubiquitously expressed in cells, localized within both the nucleus and cytoplasm, and is a zinc‐dependent class I HDAC comprising 377 amino acids.^[^ ^36^ ^]^ HDAC8 preferentially interacts with H3K9 and H3K27, deacetylating these residues and regulating the transcription of target genes implicated in the development of tumor and immune evasion.^[^ ^30^, ^37^, ^38^, ^39^ ^]^

In mouse models of hepatocellular carcinoma, HDAC8 inhibitors can remodel the epigenetic program in tumor cells and effectively restore H3K27 acetylation. This restoration promotes the infiltration of CD8^+^ T cells into tumor sites, and when paired with anti‐PD‐1 therapy, induces a robust and durable anti‐tumor response.^[^ ^30^ ^]^ Additionally, HDAC8‐specific inhibitors can ameliorate the immunosuppressive TME by modulating the transcription of the NKG2D ligand gene. This modulation enhances natural killer cell‐mediated cytotoxicity against gliomas.^[^ ^40^ ^]^

In this study, we investigated the effect of HDAC8 inhibitors on the expression of H3K27ac in CD8^+^ exhausted T cells. By predicting the binding site between H3K27ac and the transcription factor TBX21 (also known as T‐bet, a crucial regulator of T cell function^[^ ^41^ ^]^), we performed ChIP‐qPCR. Our results indicated that H3K27ac was significant enriched in the TBX21 transcription start site region. Furthermore, our animal experiments revealed that the application of HDAC8 inhibitors effectively reduced the percentage of CD8^+^ exhausted T cells in tumor sites and improved the efficacy of anti‐PD‐1 therapy.

## 4 Conclusion

Taken together, our study revealed that CK2B plays an important role in regulating T‐cell exhaustion during anti‐PD‐1 therapy for NSCLC. We also elucidated the mechanism by which CK2B upregulates TBX21 expression through HDAC8‐mediated epigenetic reprogramming, thereby preventing or even reversing CD8^+^ T‐cell exhaustion (Figure 7H). In addition, we identified CK2B as a novel biomarker for predicting the efficacy of anti‐PD‐1 therapy in NSCLC. Meanwhile, our findings establish a theoretical foundation and experimental basis for future clinical applications of shCK2B adoptive cell therapy or *CK2B* gene therapy plus anti‐PD‐1 therapy.

## 5 Experimental Section

### Cell Line and Tumor Models

This study utilized human and animal cell lines, including the human non‐small cell lung cell line (A549), and mouse lung adenocarcinoma cell lines Lewis (LLC and TC‐1). Additionally, mouse lung adenocarcinoma cell lines expressing chicken ovalbumin, TC‐1‐OVA and LLC‐OVA, were also employed. LLC and LLC‐OVA cells were cultured in complete DMEM medium supplemented with 10% serum, while TC‐1, TC‐1‐OVA, and A549 cells were cultured in complete 1640 medium with 10% serum. A549, TC‐1, and LLC cell lines were obtained from the ATCC (American Type Culture Collection).TC‐1, TC1‐OVA, LLC, and LLC‐OVA tumor cells (5 × 10^5^ or 1 × 10^6^ or as indicated) were injected subcutaneously (s.c.) into the right groin of C57BL/6J mice to establish a mouse transplantation tumor model.

### Western Blot Analyses

Protein samples were obtained from CD8^+^ T cells after treatment with CX4945 (10 µM), heterodimer (1 µg mL^−1^), thapsigargin (100 nm) or tauroursodeoxycholic acid (0.5 mm) and PCI‐34051 (20 nm) by RIPA buffer containing protease inhibitor cocktail for 30 min. These protein samples were then performed to electrophoresis and electrotransfer, and then incubated with anti‐GAPDH (Servicebio, GB11002‐100), anti‐CK2B (Proteintech, 20234‐1‐AP), anti‐HSPA5 (Proteintech, 66574‐1‐Ig), anti‐H3K27ac (Proteintech, 39 685), anti‐H3 (Proteintech, 17168‐1‐AP), anti‐HDAC5 (Proteintech, 16166‐1‐AP), anti‐HDAC8 (Proteintech, 17548‐1‐AP), β‐actin (Servicebio, GB15003), and tubulin (Servicebio, GB15140) antibodies overnight at 4 °C, and the following day were performed to exposure and image. The mean fluorescence intensity were measured by imageJ. All Western blot experiments were performed a minimum of three times for each condition.

### Immunohistochemical Staining

Paraffin tissue sections were collected from patients with NSCLC who received neoadjuvant anti‐PD‐1 mAbs or not for immunohistochemical double staining (purchased from ZSGB‐bio). The paraffin sections were deparaffinized according to protocol, and after sequentially passing through xylene, anhydrous ethanol, 95% ethanol, 85% ethanol, and 75% ethanol, and the sections were performed to antigen retrieval. Next, the sections were antigenically blocked with an endogenous peroxidase blocker and incubated overnight with antibodies against CK2B and CD8. Subsequently, the paraffin sections were double‐stained following the manufacturer's instructions. The stained paraffin sections from tumor tissues were quantitatively evaluated using a light microscope (Olympus, Japan).

### Quantitative RT‐qPCR

The suspended CD8^+^ T cells were centrifuged and then incubated with 1 mL of TRIzol reagent on ice for 30 min to extract total RNA. Subsequently, RNA extracted using chloroform was reverse transcribed into cDNA, followed by RT‐qPCR analysis of the samples' cDNA using Fast qPCR Master Mix (Low Rox) according to the manufacturer's instructions (Sangon Biotech).

The sequences of the forward and reverse primers are as follows:

**Table:** 

Gene names	Forward primers	Reverse primers
**TBX21**	CATTCCTGTCATTTACTTGGGC	CCCTTGTTGTTTGTGAGCTTTA
**TOX**	GTTGACGTGAAGACATCTCAAC	GACACAGCCATGTTTGCTATAG
**Eomes**	ATTCATCCCATCAGATTGTCCC	ACGGTTCTCTCTCGCCATTATAAT
**DDIT3**	GAGAATGAAAGGAAAGTGGCAC	ATTCACCATTCGGTCAATCAGA
**CSNK2B**	GGAGCCTGATGAAGAACTGGAAGAC	GATGCCACGGTTGGTAAGGATGTAG
**HSPA5**	CAGTTGTTACTGTACCAGCCTA	CATTTAGGCCAGCAATAGTTCC
**CSNK2A1**	CTTGGATTTCCTGGACAAACTG	GTAGAAATAGGGGGTGCTCCATT
**CSNK2A2**	TGTTAGCAAGCATGATCTTTCG	AATGTTGTCCCAGGATATCGTT
**XBP1**	CTTGTAGTTGAGAACCAGGAGT	CCCAACAGGATATCAGACTCTG

Human ACTB primers were purchased from Sangon Biotech (B661102‐0001). In the analysis of relative mRNA expression, β‐actin was utilized as a reference to normalize the samples.

### RNA Sequencing

Total RNA from CD8^+^ Tex cells treated with siRNA or CX4945 was obtained using the TRziol. RNA was initially quantified using a NanoDrop 2000, and an Agilent 2100/4200 Bioanalyzer was used to read the quality of the RNA samples and accurately quantify the concentration. The samples were then processed for RNA‐seq using an Illumina NovaSeq 6000. Read counts for all samples were normalized using the calcNormFactors function of the R/Bioconductor software package. Differential expression analysis between CD8^+^ Tex cells treated with siRNA or CX4945 and CD8^+^ Tex cells from control group was performed by the negative binomial distribution model and poisson distribution model implemented in edgeR. Genes with a *P <* 0.05 were considered significant after multiple testing with Benjamini‐Hochberg correction.

### Chromatin Immunoprecipitation (ChIP)‐qPCR

ChIP assay was performed using the SimpleChIP Enzymatic Chromatin IP Kit (Thermo Fisher Scientific, 26 157). Sheared chromatin was immunoprecipitated with anti‐H3K27ac antibody (Active Motif, 30 985) or rabbit IgG isotype antibody (Cell Signaling Technology, 2729S). Quantitative PCR analysis of purified DNA was performed with pre‐validated primers (synthesized by Sangon Biotech Inc) that detect the H3K27ac binding sequence in the TBX21 promoter.

### Preparation of Single‐Cell Suspensions and Flow Assay

The single‐cell suspensions of mouse spleen and draining lymph nodes were obtained via mechanical grinding. Mouse transplant tumor tissues were enzymatically digested with collagenase IV and DNA hydrolase I at (1 mg mL^−1^ and 100 µg mL^−1^), respectively, and filtered through a 70 µm cell filter to remove impurities. Subsequent erythrocyte lysis resulted in a single‐cell suspension prepared for flow cytometry.

For surface antigens staining, cells were first stained for viability using the LIVE/DEAD™ Fixable Dead Cell Staining Kit (Invitrogen™) before proceeding to antibody labeling and flow cytometric analysis. For intracellular staining: 1) For cytokine staining, cells were activated by in vitro stimulation prior to surface antigens staining. After stimulation, cells were fixation and permeabilization (BD Cytofix/Cytoperm kit, BD Biosciences) and then stained for intracellular antigens; 2) For non‐cytokine staining, the experimental procedure followed the steps in 1, excluding the in vitro activation step. Flow cytometric analysis was performed using a BD or Beckman machine, and data were analyzed by FlowJo V10 software.

### Antibodies for Flow Cytometry

**Table:** 

**Antibodies**	**Identifier**	**Source**
Anti‐human CD3 Antibody	317 306	Biolegend
Anti‐human CD8a Antibody	300 924	Biolegend
Anti‐human CD366 (Tim‐3) Antibody	345 034	Biolegend
Anti‐human CD279 (PD‐1) Antibody	329 922	Biolegend
Anti‐human CD279 (PD‐1) Antibody	329 908	Biolegend
Anti‐TCF1 (TCF7) Antibody	655 208	Biolegend
Anti‐IFN‐γ Antibody	502 509	Biolegend
Anti‐T‐bet Antibody	644 808	Biolegend
Goat anti‐mouse IgG (minimal x‐reactivity) Antibody	405 316	Biolegend
Anti‐mouse CD3 Antibody	100 204	Biolegend
Anti‐mouse CD3 Antibody	100 218	Biolegend
Anti‐mouse CD8a Antibody	100 722	Biolegend
Anti‐mouse CD45 Antibody	103 131	Biolegend
Anti‐mouse CD366 (Tim‐3) Antibody	134 014	Biolegend
Anti‐mouse CD366 (Tim‐3) Antibody	119 748	Biolegend
Anti‐mouse CD279 (PD‐1) Antibody	109 112	Biolegend
Anti‐mouse CD279 (PD‐1) Antibody	135 223	Biolegend
Anti‐human/mouse Granzyme B Antibody	515 406	Biolegend
Anti‐mouse IFN‐γ Antibody	505 846	Biolegend
Anti‐mouse IFN‐γ Antibody	505 808	Biolegend
Anti‐mouse CD45.1	110 705	Biolegend
Anti‐mouse CD45.2	109 824	Biolegend
Goat anti rabbit IgG(H+L)	A0468	Beyotime
Zombie Violet™ Fixable Viability Kit	423 113	Biolegend
Goat Anti‐Mouse IgG H&L	ab150115	Abcam
Zombie NIR™ Fixable Viability Kit	423 105	Biolegend
Zombie Green™ Fixable Viability Kit	423 112	Biolegend

### Single‐Cell RNA Sequencing

Lymphocyte suspensions prepared from tumor tissue of 12 patients diagnosed with stage IIIA NSCLC were included in the single‐cell sequencing analysis, consisting of 4 treatment‐naïve patients and 8 patients treated with neoadjuvant pembrolizumab and chemotherapy. Cell Ranger 3.1.0 was employed to align and quantify the scRNA‐seq data against the GRCh38 human reference genome. Subsequently, a gene expression matrix was generated and filtered cell identification barcodes using Seurat 3.2.1 to exclude dead cells and artifacts.

Specifically, cells were filtered based on criteria: <200 or >6000 genes detected per cell, UMI <1000, or more than 10% mitochondrial genes. To address batch effects between samples, the FindIntegrationAnchors function was utilized from the Seurat package, employing the MNN algorithm.^[^ ^42^ ^]^ A total of 10127 CD8^+^ T cells were included in the final analysis.

### Preparation of WT OT‐I, ShCK2B OT‐I T Cells, and Human CD8^+^ T Cells

PBMCs from healthy volunteers were stimulated and activated with anti‐human CD3/CD28 antibodies (STEM CELL) in medium containing human IL‐2 for 3 days. Activated CD8^+^ T cells were isolated using either magnetic beads (Miltenyi Biotec) or flow sorting technology for subsequent experiments.

The spleens from OT‐1 mice were collected and mechanically fragmented, followed by filtration through a 70 µm filter to remove impurities. Erythrocytes were lysed by adding ACK lysis buffer to the cell suspension for 5 min at 25 °C. The splenocytes were then washed twice with pre‐cooled complete RPMI medium (10% FBS, 1% penicillin/streptomycin, 1% HEPES, 1% sodium pyruvate, 0.1% 2‐mercaptoethanol).

Next, the splenocytes were resuspended in complete RPMI medium at a density of 1.5‐2 × 10^6^ cells mL^−1^ and supplemented with IL‐2 (10 ng mL^−1^) and OVA257‐264 peptide (1 µm, GenScript). After 3 days of culture, live cells were enriched using Ficoll's solution and further cultured at a density of 1.0–1.5 × 10^6^ cells mL^−1^ in complete RPMI medium for an additional 2 days to obtain activated CD8^+^ T cells with a purity exceeding 95%. These activated CD8^+^ T cells would be utilized in mouse adoptive cell transfer (ACT) experiments or western blot analyses.

For retroviral transduction, mouse CD8^+^ T cells isolated from spleens were activated with Dynabeads magnetic beads (1:1 bead‐to‐cell ratio, ThermoFisher Scientific) for 48 h. The stimulated T cells were then mixed with retroviruses in 24‐well plates precoated with recombinant human fibronectin fragment (25 µg mL^−1^, Solarbio) according to the manufacturer's instructions.

For siRNA transfection, human CD8^+^ T cells were first activated by plate‐conjugated anti‐CD3/CD28 mAbs, followed by transfection with siRNA using the manufacturer's recommended transfection reagent protocol.

### In Vitro Cytotoxic Assays

According to previous literature, CIK cells were derived from PBMCs expanded in vitro for 14 days.^[^ ^43^ ^]^ Post‐treatment with siRNA or CX4945, CIK cells were co‐cultured with A549 cells (after affixation 24 h) for 24 h in 96‐well plates. Cytotoxicity assays were performed by measuring the release of LDH (lactate dehydrogenase) in the culture supernatant and optical density values generated by specific lysis of target cells.

### In Vitro Induction of CD8^+^ T Cell Exhaustion

According to previous study, activated CD8^+^ T cells (day 7 of culture) were induced exhaustion for 2 days with dimerized anti‐CD3 antibody [prepared by mixing anti‐CD3 antibody (Biolegend, 317 302) with goat anti‐rat IgG (Thermo Fisher Scientific, A16068) at a 2:1 molar ratio] in complete RPMI medium supplemented with IL‐2 (10 ng mL^−1^).^[^ ^21^ ^]^ Flow cytometry characterized harvested cells based on surface inhibitory receptor expression (PD‐1 and Tim‐3). Flow cytometry was used to analyze live CD8^+^ T cells, and a subset of PD‐1^+^Tim‐3^+^CD8^+^ T cells was sorted for subsequent in vitro co‐culture assays.

### Co‐culture Of CD8^+^ T Cells and Tumor Cells In Vitro

A549 cells were cultured in vitro and expanded. The expanded cells were then trypsinized and seeded at a density of 2 × 10^5^ cells per well in 6‐well plates containing complete 1640 medium supplemented with 10% FBS. After 2 days of incubation at 37 °C, the culture medium was removed. Activated CD8^+^ T cells or PD‐1^+^CD8^+^ T cells in suspension were subsequently added to the co‐culture system at a ratio of 5:1 (T cells to tumor cells). After an additional 2 days of co‐culture, CD8^+^ T cells were isolated using Ficoll's solution and analyzed by flow cytometry.

### In Vivo Animal Work

Five to six‐week‐old female C57BL/6J mice were purchased from SPF, Inc, and subcutaneously injected with 1 × 10^6^ or 5 × 10^5^ LLC, LLC‐OVA, TC‐1, or TC‐1‐OVA cells/100 µL PBS establish a transplantation tumor model. Drug interventions with CX4945 (10 mg kg^−1^ perday, per mouse), PCI‐34051 (200 µg per day, per mouse), chloroquine (0.2 mg/per mouse/day) and anti‐PD‐1 mAbs (150 µg/twice a week, per mouse) were initiated when the transplanted tumor volume reached approximately 50–100 mm^3^ (Tumor volume was measured with electronic vernier calipers 3 times per week). In adoptive transfer experiments using OT‐1 T cells, as described in previous literature, lymphodepletion was induced with intraperitoneal administration of cyclophosphamide (300 mg kg^−1^) one day prior to adoptive transfer of OT‐1 CD8^+^ T cells.^[^ ^44^ ^]^ For other administration schedules, reference was made to a schematic diagram. According to a previous study, tumor volume was measured as follows: V = width × width × length/0.5.^[^ ^45^ ^]^ The survival endpoint in mice was set, defined as when mean LLC or LLC‐OVA tumor volume exceeded 1500 mm^3^ (mean TC‐1 or TC‐1‐OVA tumor volume exceeded 2000 mm^3^). The fold change of tumor volume (FCTV) was calculated as follows: FCTV  =  Ve /V0, where V0 is the tumor volumes measured at first treatment and Ve is the tumor volume measured each time during treatment. a FCTG value < 4 was recognized as effectively controlled tumor growth.

### Clinical Data Analysis

A total of 49 patients were enrolled in the histochemical double staining experiment, comprising 20 patients with NSCLC treatment with anti‐PD‐1 neoadjuvant therapy and 29 patients who did not. Inclusion criteria included patients with complete clinical data and follow‐up, who underwent surgical therapy at Tianjin Medical University Cancer Hospital and had stored paraffin tissue sections available.

Additionally, a retrospective study included 10 patients with advanced NSCLC treated CIK cell therapy plus anti‐PD‐1 mAbs. Inclusion criteria for this study were patients who received at least two sessions of CIK cell therapy and were evaluated for efficacy, with PBMC samples obtained before and after treatment. All clinical data such as age at diagnosis, pathology type, gender, smoking history, best efficacy evaluation, clinical stage, and TNM stage were recorded and analyzed by SPSS.

### Statistical Analysis

The statistical analyses for this study were performed by GraphPad Prism 9 software. Data were presented as mean ± s.d. among groups unless otherwise noted. Comparisons between two groups were assessed using unpaired Student's t‐tests, while comparisons among multiple groups were performed by ANOVA and Tukey's test. Survival data relevant to clinical outcomes were evaluated using the log‐rank test. Differences were deemed statistically nonsignificant (NS) when the *P* > 0.05.

### Ethics Approval And Consent To Participate

Animal experiments in this study were approved by the Animal Ethics and Welfare Committee of Tianjin Medical University (TMUaMEC2022030). All animal experiments were conducted in strict accordance with approved protocols and adhered to relevant guidelines and regulations, none of the mouse transplantation tumors exceeded 2 cm in diameter (According to the Ethics Committee, tumors in mice weighing 25 g or less were not allowed to exceed 2 cm in diameter). The clinical studies in this study were approved by the Ethics Committee of Tianjin Medical University Cancer Hospital (No: E2019091) and informed consent was obtained from all subjects.

## Conflict of Interest

The authors declare no conflict of interest.

## Author Contributions

S.L., S.M., G.L., L.H., and Y.G. contributed equally to this work. S.L. participated in the design of this study, performed most of the experiments, and wrote the manuscript. S.M., L.H., and G.L. performed in vivo animal experiments and some in vitro experiments. Y.G. and L.L. contributed to the design and conception of this study, and the analysis and organization of the data. Y.M., W.Y., and T.L. contributed to the preparation of animal specimens, and the detection by flow cytometry. L.Z., Z.Y., P.J., L.Y., and Z.Y. contributed to the collection of clinical specimens and clinical data. Q.S. and X.R. guided the design of the study and revised this manuscript.

## Supporting information



Supporting Information

## Data Availability

The data that support the findings of this study are available from the corresponding author upon reasonable request.
