# Supplementary material for: CK2B Induces CD8+ T‐Cell Exhaustion through HDAC8‐Mediated Epigenetic Reprogramming to Limit the Efficacy of Anti‐PD‐1 Therapy in Non‐Small‐Cell Lung Cancer
Source: Adv Sci (Weinh). 2025 Feb 27;12(16):2411053. doi: 10.1002/advs.202411053 (PMC12021095; doi:10.1002/advs.202411053)
Supplement: Supplementary file 1 — Supporting Information [file ADVS-12-2411053-s001.doc]

**CK2B Induces CD8^+^ T-Cell Exhaustion Through HDAC8-Mediated Epigenetic Reprogramming To Limit The Efficacy Of anti-PD-1 Therapy In Non-Small-Cell Lung Cancer**

Shaochuan Liu, Shiya Ma, Gen Liu, Lingjie Hou, Yong Guan, Liang Liu, Yuan Meng, Wenwen Yu, Ting Liu, Li Zhou, Zhiyong Yuan, Shuju Pang, Siyuan Zhang, Junyi Li,, Xiubao Ren, Qian Sun.

**Supplementary information includes 13 figures and 1 table**


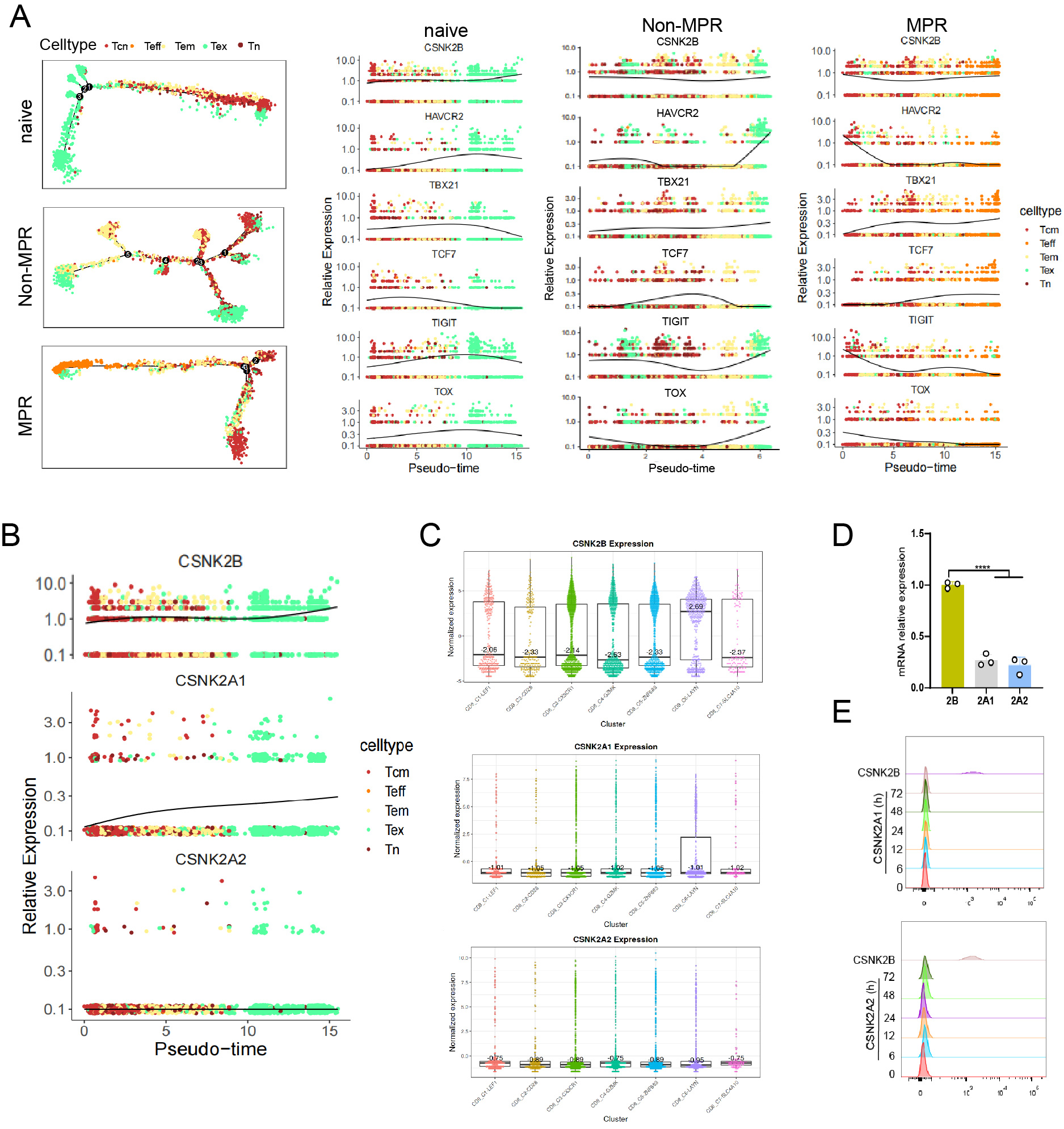


**Fig S1: (A)** Trajectory analysis of *CSNK2B*, *HAVCR2*, *TBX21*, *TCF-7*, *TIGIT*, and *TOX* from naïve, Non-MPR, and MPR three groups. **(B)** Trajectory analysis of *CSNK2B, CSNK2A1,* and *CSNK2A2* from naïve group. **(C)** Expression levels of *CSNK2B* in various CD8^+^ T-cell subsets (the purple represents CD8^+^ Tex cells) in a public NSCLC scRNA-seq database (http://lung.cancer-pku.cn/index.php). **(D)** mRNA relative expression of *CSNK2B, CSNK2A1,* and *CSNK2A2* in CD8^+^ Tex cells. **(E)** Expression levels of CSNK2A1, CSNK2A2, and CSNK2B in an induced CD8^+^ T cell exhaustion model. **P* < 0.05, ***P* < 0.01, ****P* < 0.001, and *****P* < 0.0001.


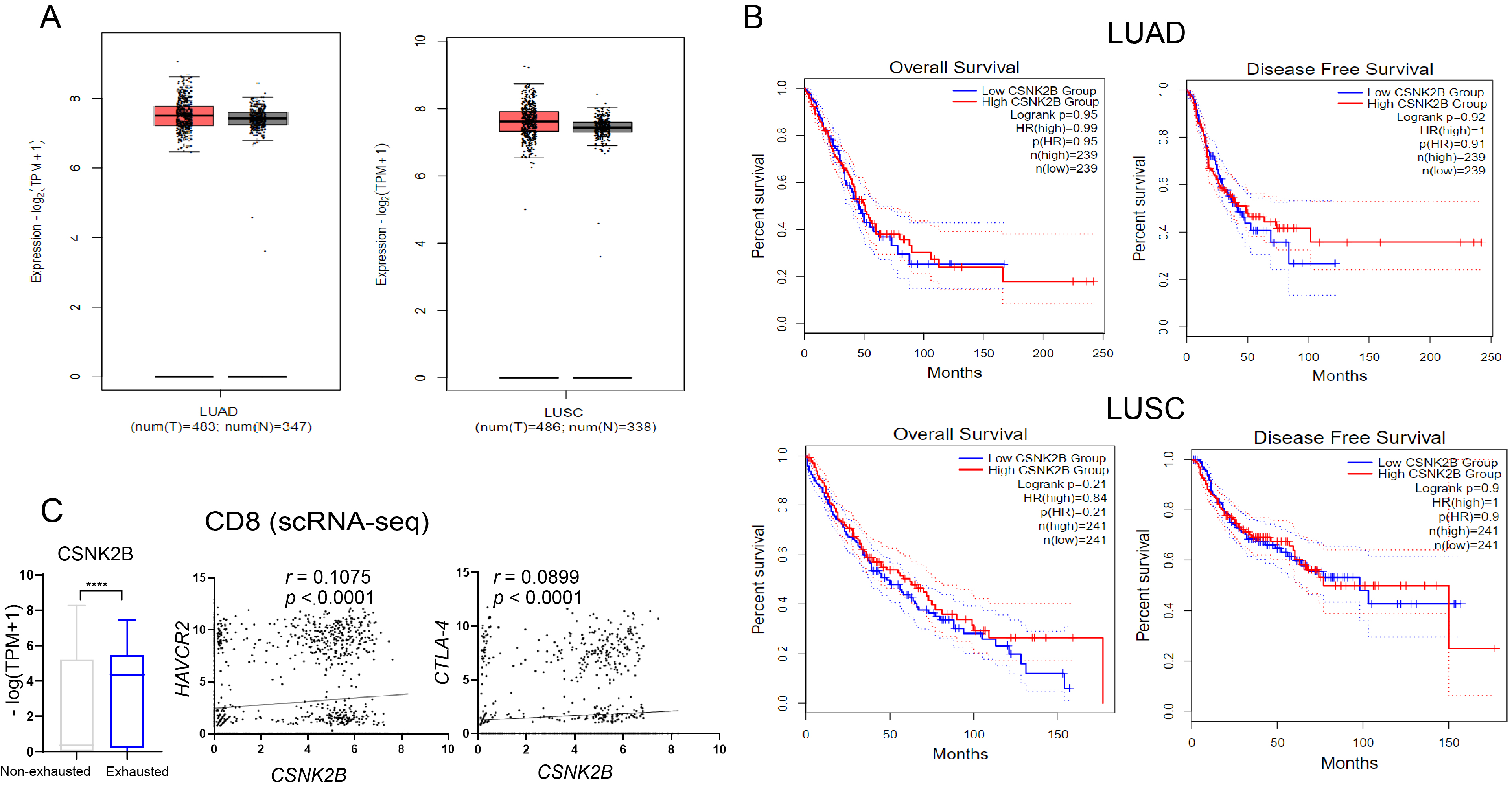


**Fig S2:** **(A)** Expression level of *CSNK2B* in LUSC and LUAD compared to paracancerous tissues (GEPIA databases). **(B)** Relationship between high and low *CSNK2B* expression in LUAD and LUSC and patients' survival prognosis. **(C)** Public scRNA-seq data show that *CSNK2B* expression levels in CD8^+^ Tex cells and non-Tex cells, and correlation with *HAVCR2* and *CTLA4.* *****P* < 0.0001 (Pearson's test and Student's t test).


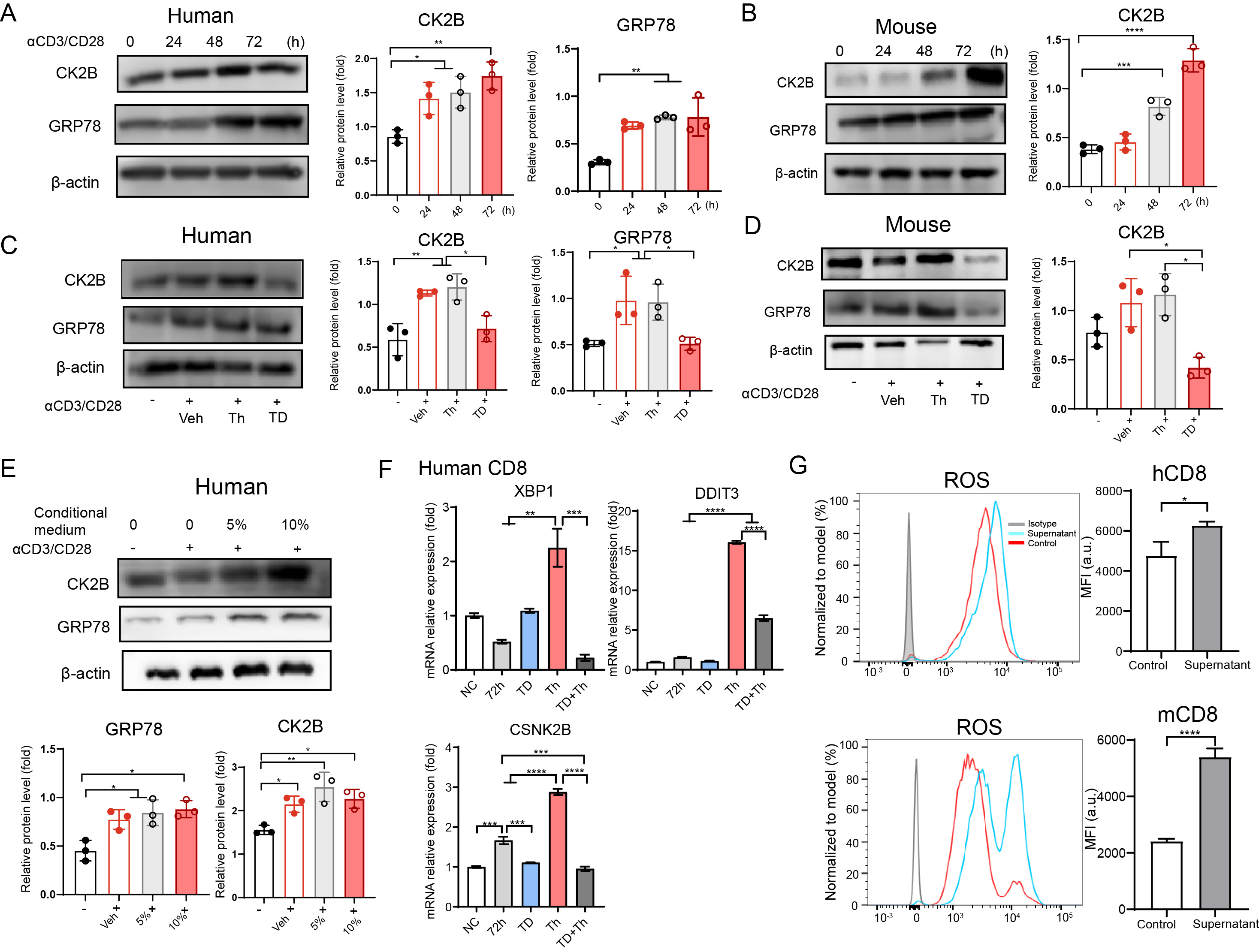


**Fig S3:** Time-dependent induction of CK2B and GPR78 in human **(A, left)** and murine **(B, left)** CD8^+^ T cells in *vitro*. CD8^+^ T cells were stimulated with αCD3/CD28 and cellular proteins were collected at the indicated time points (0–72 h); western blot analysis of the protein expression levels of CK2B, GPR78, and β-actin in human **(A, right)** and murine **(B, right)** CD8^+^ T cells. **(C-D)** Western blot analysis of the protein expression levels of CK2B, GPR78, and β-actin in human and murine CD8^+^ T cells following anti-CD3/CD28 stimulation by treatment with thapsigargin (100 nM, after 48 h) or tauroursodeoxycholic acid (0.5 mM, at time 0). **(E)** Western blot analysis of the protein expression levels of CK2B, GPR78, and β-actin in human CD8^+^ T cells treated with tumor conditional medium (at indicated concentrations) and αCD3/CD28. **(F)** mRNA expression of *XBP1*, *CSNK2B*, and *DDIT3* in human CD8^+^ T cells treated with αCD3/CD28, thapsigargin, and tauroursodeoxycholic acid (Data are expressed as mean ± s.e.m). **(G)** Flow cytometric detection of ROS expression levels in CD8^+^ T cells stimulated with 10% tumor supernatants (A549 or LLC) for 24h; thapsigargin: Th; tauroursodeoxycholic acid: TD. **P* < 0.05, ***P* < 0.01, ****P* < 0.001, and *****P* < 0.0001 (Student's t test).


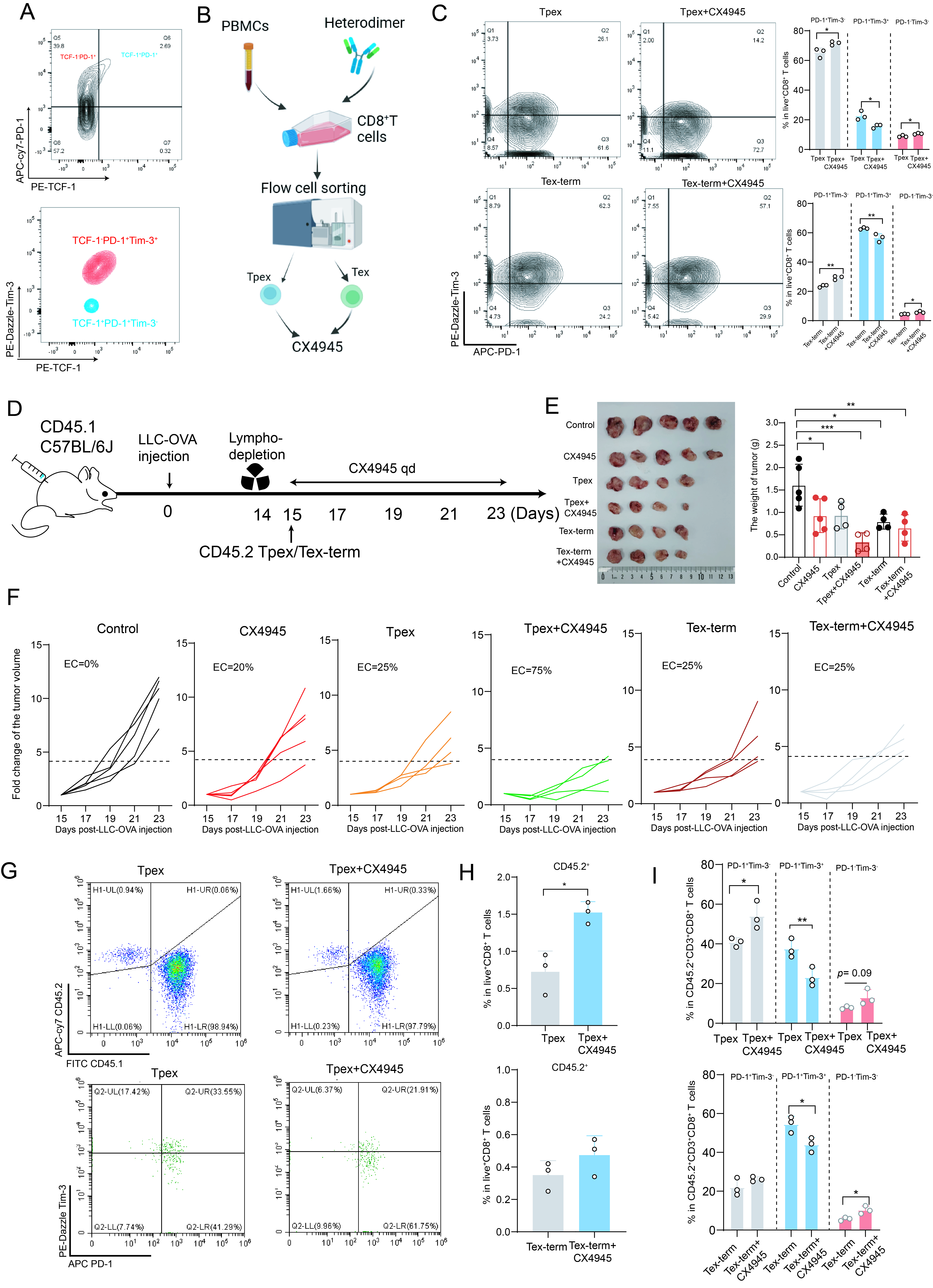


**Fig S4: (A)** Typical flow cytometry pictures showing Tpex cells and Tex-term cells. **(B)** Schematic diagram illustration of lymphocyte-induced exhaustion followed by sorting into precursor and terminally exhausted T cells for subsequent experiments; **(C)** sorted terminally exhausted T cells and precursor exhausted T cells were treated with CX4945 for 24 h in *vitro* experiments, respectively, and then subjected to changes in expression of the relevant subpopulations. **(D)** On day 0, CD45.1^+^ C57BL/6J mice were inoculated with LLC-OVA tumor cells. On day 14, the mice were sublethally lymphodepleted and received PBS (n=5), CX4945 (n=5), adoptive activated OT1 CD45.2^+^CD8^+^ Tpex cells (n = 4), OT1 CD45.2^+^CD8^+^ Tpex cells plus CX4945 (n = 4), OT-1 CD45.2^+^CD8^+^ Tex-term cells (n = 4) or adoptive activated OT-1 CD45.2^+^CD8^+^ Tex-term cells plus CX4945 (n = 4). On day 23, mice were sacrificed for flow cytometry analyses of TILs. (**E**) Image of the collected mouse tumors (left) and a statistical graph of the weight of the tumors (right). (**F**) Individual change curve of tumor volume in mice (fold change of the tumor volume not exceeding 4 was defined as tumors that were effectively controlled to be recorded). (**G**) Representative flow cytometry graphs showing the adoptive CD45.2^+^CD8^+^ OT-1 cells and T-cell exhaustion infiltrated within the tumor sites among Tpex cells and Tpex cells plus CX4945 groups. (**H**) The statistical charts of the frequencies of adoptive CD45.2^+^CD8^+^ OT-1 cells infiltrated within the tumor sites. (**I**) Flow cytometry analysis of the expression levels of PD-1 and Tim-3 in CD45.2^+^CD8^+^ Tpex cells or Tex-term cells infiltrated within the tumor sites among different treatment groups. **P* < 0.05, ***P* < 0.01, ****P* < 0.001, and *****P* < 0.0001 (one-way ANOVA or Student's t test).


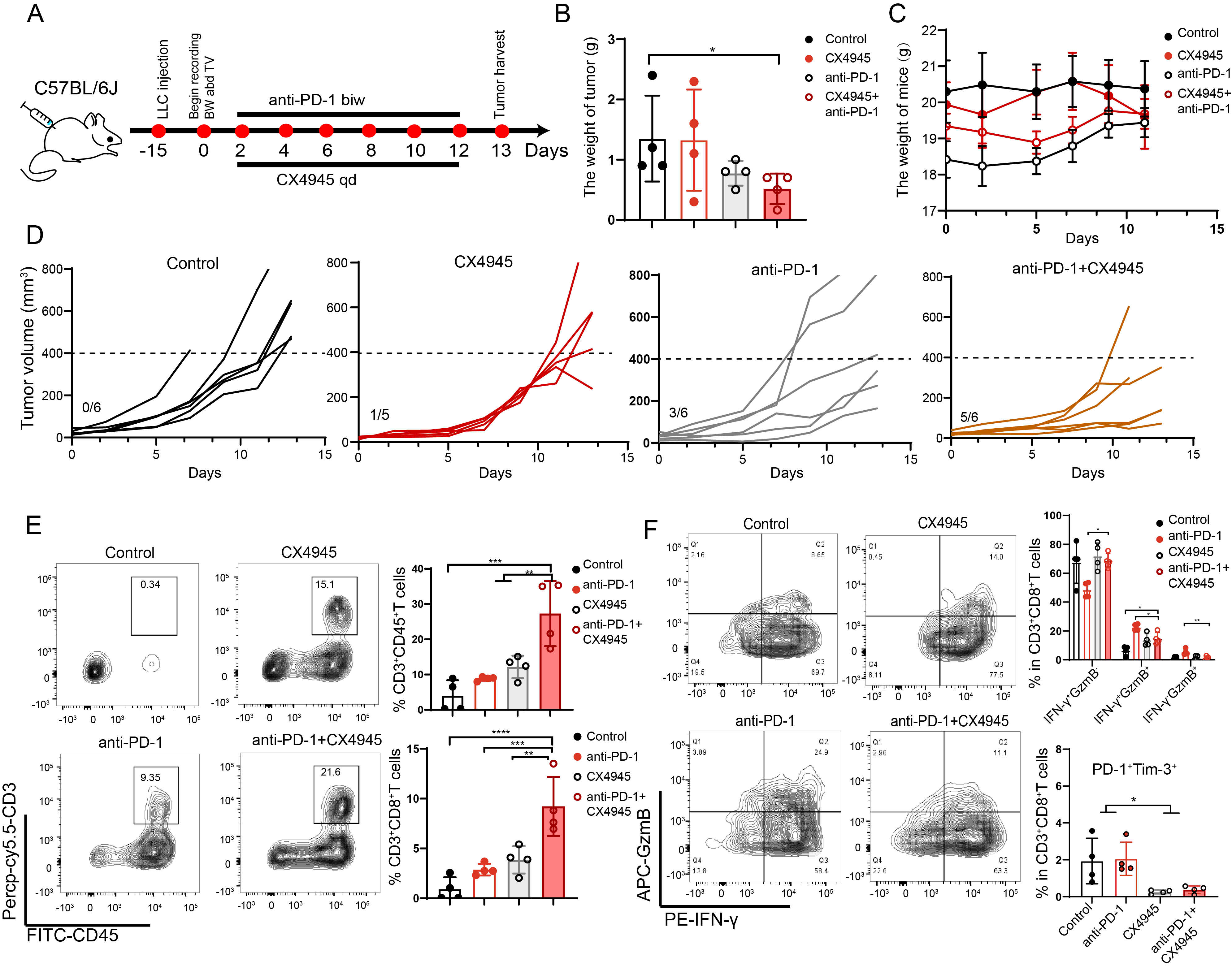


**Fig S5:** C57BL/6J mice were inoculated with LLC tumor cells. On day 2, the mice were administered with CX4945 (10 mg/kg/day), anti-PD-1 mAbs (150 μg/per mouse, twice a week), or PBS control. On day 13, mice were sacrificed for flow cytometry analyses of T cells. **(A)** Diagram of subcutaneous transplantation tumor model construction and drug administration pattern. **(B)** Statistical graph of tumor weight in mice (n=4). **(C)** Body weight–time change curve in mice (data are expressed as mean ± s.e.m). **(D)** Individual change curve of tumor volume in mice (tumor volume of mice not exceeding 400 mm^3^ was defined as tumors that were effectively controlled to be recorded). **(E)** Representative flow cytometry graphs showing the frequencies of CD3^+^CD45^+^ T and CD8^+^ T cells infiltrated within the tumors (left) and statistical plots (right). **(F)** Flow cytogram showing representative IFN-γ and GzmB expression (Left), and flow cytometry analysis of the expression levels of IFN-γ and GzmB, as well as PD-1 and Tim-3, in tumor-infiltrating CD8^+^ T cells among different treatment groups (right). BW: body weight of mice; TV: tumor volume; biw: twice a week. **P* < 0.05, ***P* < 0.01, ****P* < 0.001, and *****P* < 0.0001 (one-way ANOVA or Student's t test).


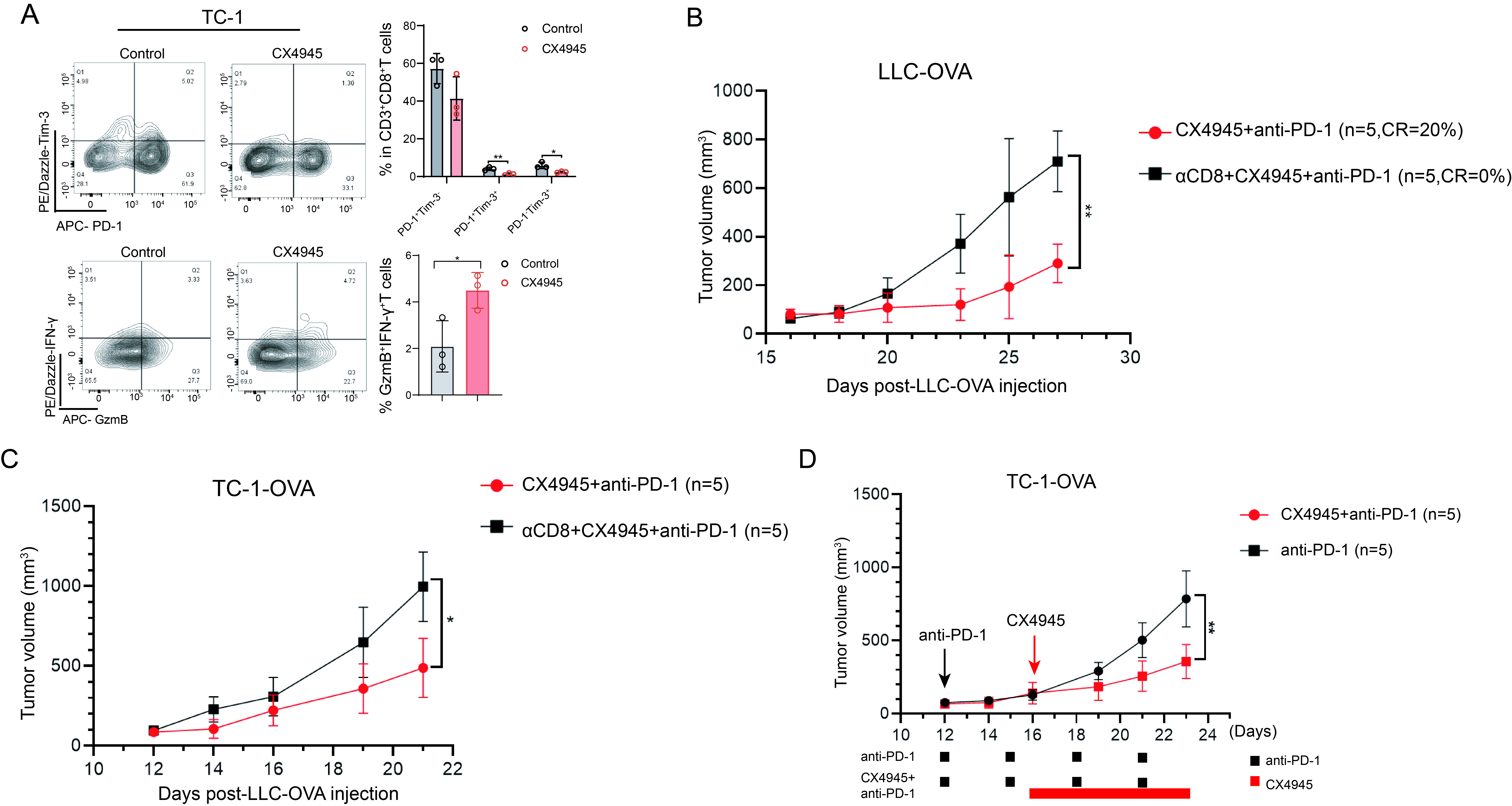


**Fig S6: (A)** Flow cytometry analysis of the expression levels of IFN-γ and GzmB, as well as PD-1 and Tim-3, in the tumor-infiltrating CD8^+^ T cells in TC-1 transplantation tumor mouse model. C57BL/6J mice were inoculated with LLC-OVA tumor cells. On day 14, the mice were administered with αCD8 (200 μg/per mouse, twice a week). On day 16, the mice were administered with CX4945 (10 mg/kg/day), anti-PD-1 mAbs (150 μg/per mouse, twice a week), or PBS control; **(B)** tumor volume–time change curve in mice with LLC-OVA tumor cells. C57BL/6J mice were inoculated with TC-1-OVA tumor cells. On day 10, the mice were administered with αCD8 (200 μg/ per mouse, twice a week). On day 12, the mice were administered with CX4945 (10 mg/kg/day), anti-PD-1 mAbs (150 μg/per mouse, twice a week), or PBS control; **(C)** tumor volume–time change curve in mice with TC-1-OVA tumor cells. C57BL/6J mice were inoculated with TC-1-OVA tumor cells. On day 12, the mice were administered with anti-PD-1 mAbs (150 μg/per mouse, twice a week). On day 16, the mice were administered with CX4945 (10 mg/kg/day); **(D)** tumor volume–time change curve in mice with TC-1-OVA tumor cells, and at the bottom of the curve represent the administration of anti-PD-1 mAbs or CX4945 at different time points, respectively. **P* < 0.05, ***P* < 0.01, ****P* < 0.001, and *****P* < 0.0001 (Student's t test).


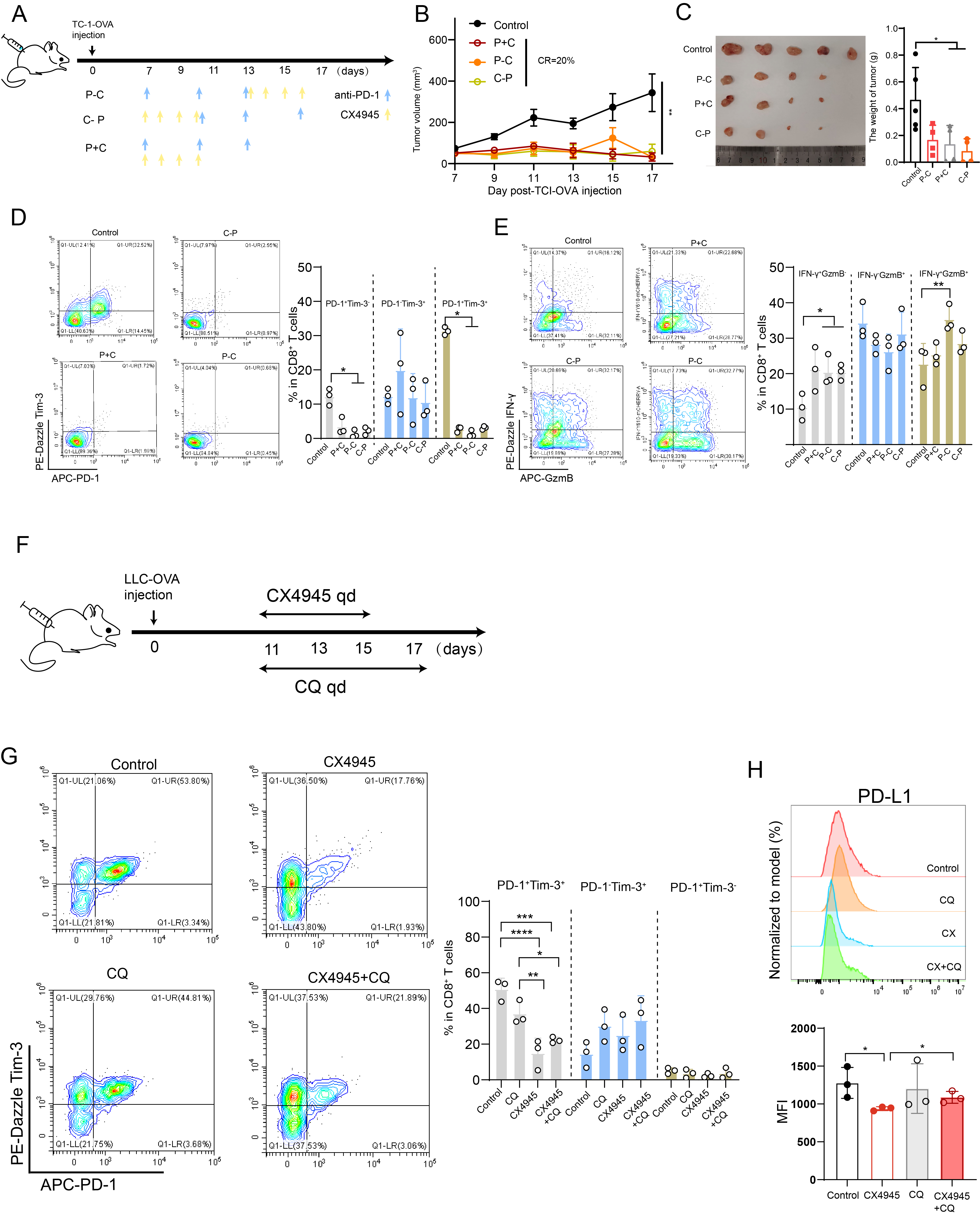


**Fig S7:** C57BL/6J mice were inoculated with TC-1-OVA tumor cells. On day 7, the mice were administered with CX4945 (10 mg/kg/day), anti-PD-1 mAbs (150 μg/per mouse, twice a week), or PBS control. On day 17, mice were sacrificed for flow cytometry analyses of T cells. **(A)** Diagram of subcutaneous transplantation tumor model construction and drug administration pattern (5 mice per group). **(B)** Tumor volume–time change curve in mice with TC-1-OVA tumor cells (data are expressed as mean ± s.e.m). **(C)** Image of the collected mouse tumors (left) and a statistical graph of the weight of the tumors (right). **(D)** Flow cytogram showing representative PD-1 and Tim-3 expression (Left), and flow cytometry analysis of the expression levels of PD-1 and Tim-3 in CD8^+^ T cells among different treatment groups (right). **(E)** Flow cytogram showing representative IFN-γ and GzmB expression (Left), and flow cytometry analysis of the expression levels of IFN-γ and GzmB in CD8^+^ T cells among different treatment groups (right). C57BL/6J mice were inoculated with LLC-OVA tumor cells. On day 11, the mice were administered with CX4945 (10 mg/kg/day), chloroquine (CQ) (0.2 mg/per mice/day), or PBS control. On day 17, mice were sacrificed for flow cytometry analyses of T cells. **(F)** Diagram of subcutaneous transplantation tumor model construction and drug administration pattern. **(G)** Flow cytogram showing representative PD-1 and Tim-3 expression (Left), and flow cytometry analysis of the expression levels of PD-1 and Tim-3 in CD8^+^ T cells among different treatment groups (right). (**H**) Mean fluorescence intensity (MFI) of PD-L1 expression was calculated for CD45-negative cell populations. CQ: chloroquine; C+P: CX4945 plus anti-PD-1 mAbs; C-P: administration of CX4945 followed by anti-PD-1 mAbs; P-C: administration of anti-PD-1 mAbs followed by CX4945.**P* < 0.05, ***P* < 0.01, ****P* < 0.001, and *****P* < 0.0001 (one-way ANOVA or Student's t test).


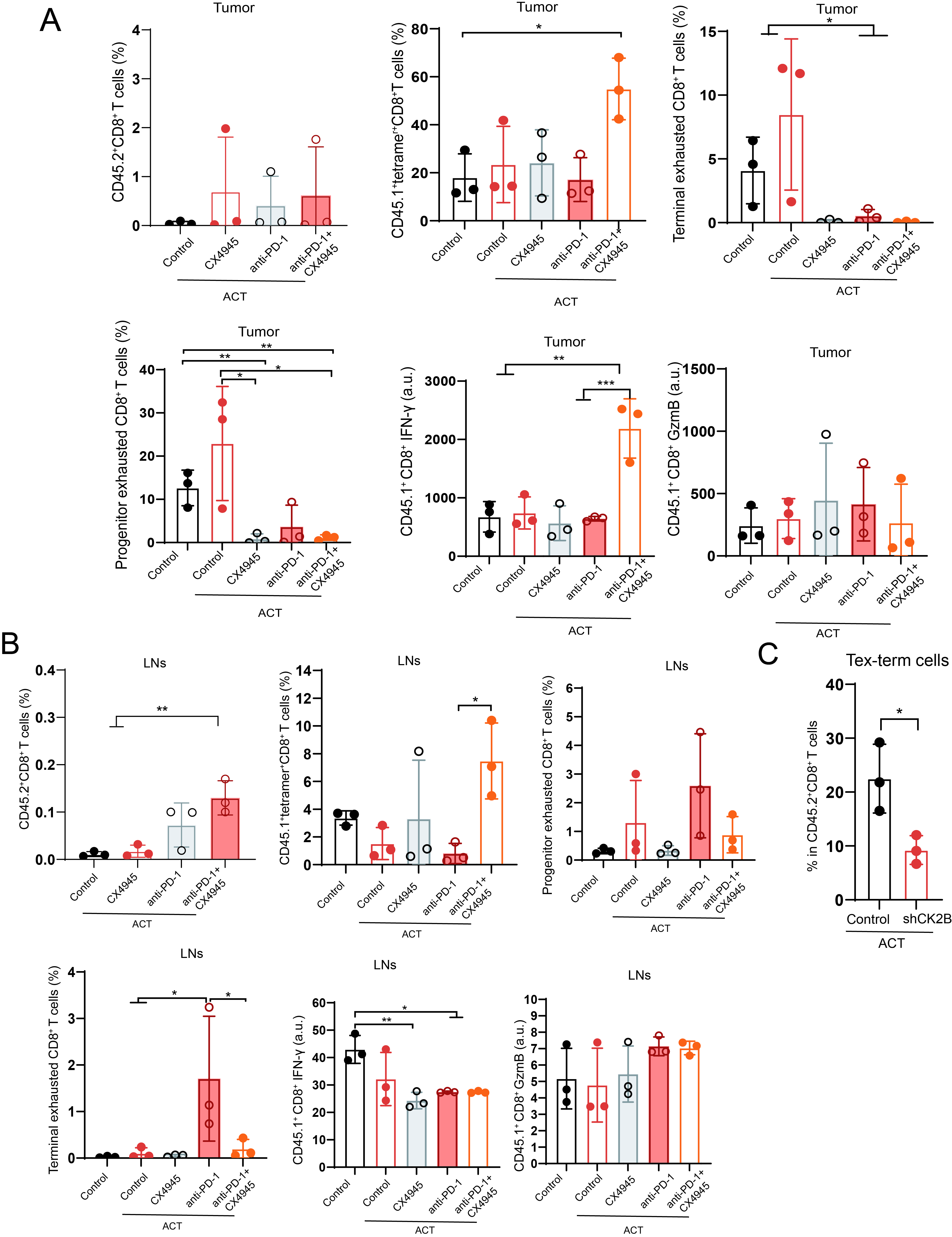


**Fig S8: (A)** Flow cytometry analysis of the percentage of adoptive CD45.2^+^CD8^+^ T cells and CD45.1^+^CD8^+^ T cell exhaustion from different groups in tumor sites. **(B)** Flow cytometry analysis of the percentage of adoptive CD45.2^+^CD8^+^ T cells and CD45.1^+^CD8^+^ T cell exhaustion from different groups in draining LNs. **(C)** Flow cytometry analysis of the percentage of adoptive CD45.2^+^CD8^+^ Tex-term cells in tumor sites from control group and shCK2B group. LNs: lymph nodes; ACT: adoptive cellular therapy. **P* < 0.05, ***P* < 0.01, ****P* < 0.001, and *****P* < 0.0001 (one-way ANOVA or Student's t test).


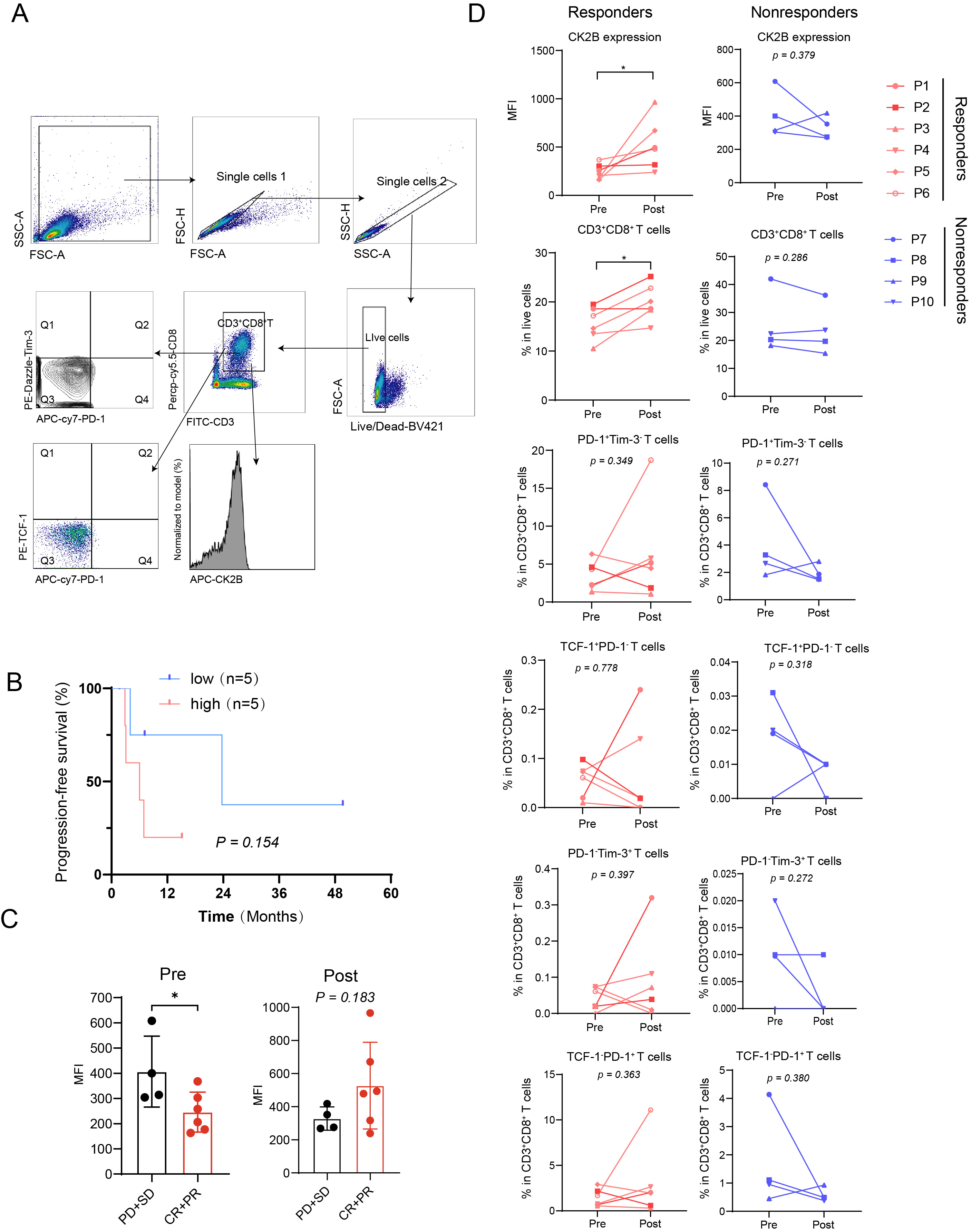


**Fig S9: (A)** Representative flow cytometry gating strategy were used to analyze PBMCs from NSCLC patients receiving CIK cell therapy in combination with anti-PD-1 mAbs plus chemotherapy. **(B)** Kaplan-Meier estimates of progression-free survival in the two groups (CK2B high expression group and CK2B low expression group). **(C)** Flow cytometry examination of CK2B expression in CD3^+^CD8^+^ T cells from PR+CR group and PD+SD group before and after treatment with CIK cells combined with chemotherapy plus anti-PD-1 mAbs, respectively. (**D**) Flow cytometry examined CK2B expression and the percentages of CD3^+^CD8^+^ T cells, PD-1^+^Tim-3^-^ T cells, PD-1^-^Tim-3^+^ T cells, TCF-1^+^PD-1^-^ T cells, and TCF-1^-^PD-1^+^ T cells from the nonresponders or responders before and after treatment. CR: complete response; PR: partial response; SD: stable disease; PD: progression disease; **P* < 0.05, ***P* < 0.01, ****P* < 0.001, and *****P* < 0.0001 (Log-rank test, Wilcoxon test or Student's t test).


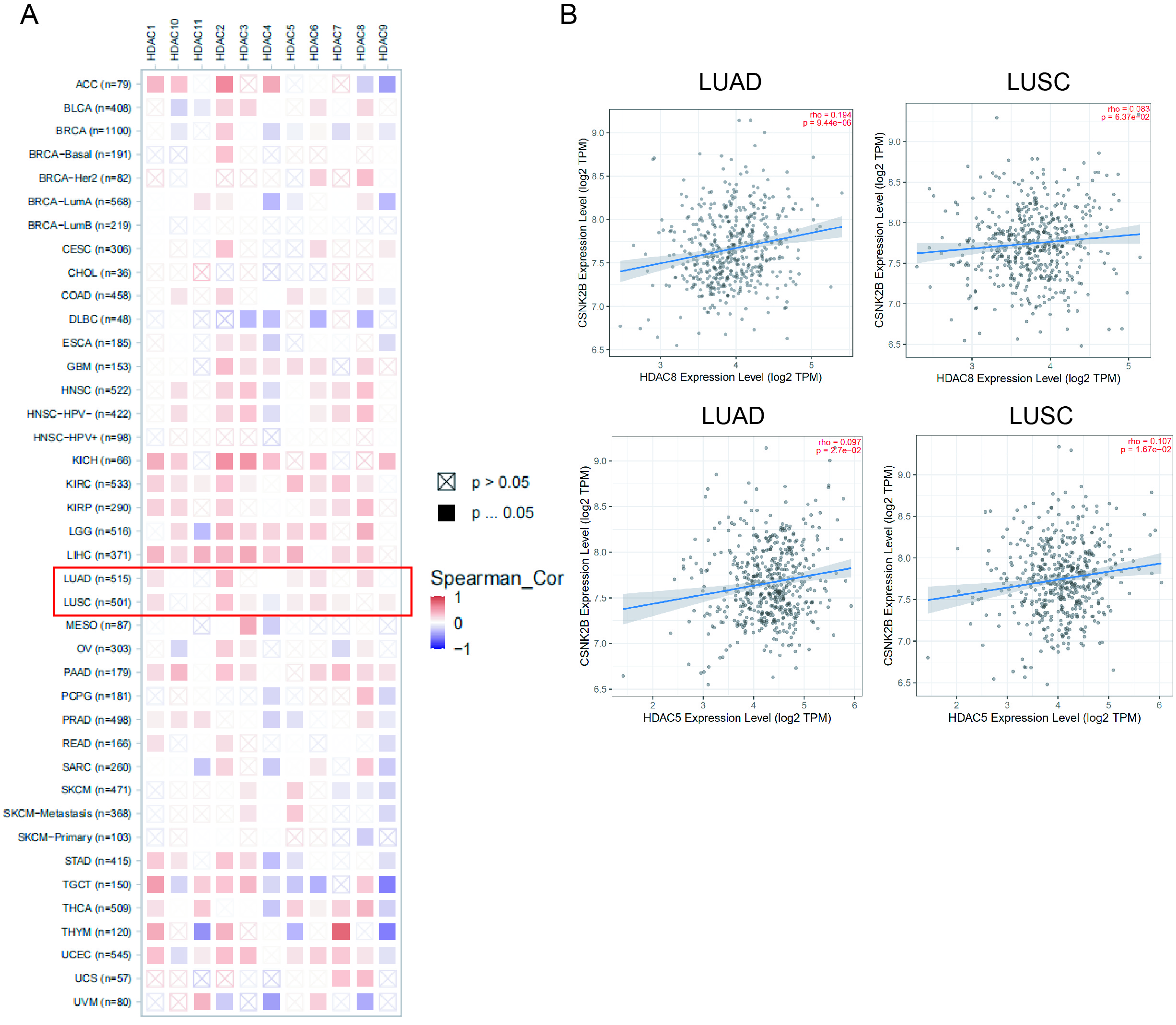


**Fig S10:** **(A)** Public database TIMER 2.0 was used to analyze the correlation between *CSNK2B* expression and HDAC family related genes. (**B**) Correlation of *CSNK2B* expression with *HDAC5* and *HDAC8* expression in LUAD and LUSC in the TIMER2.0 database.


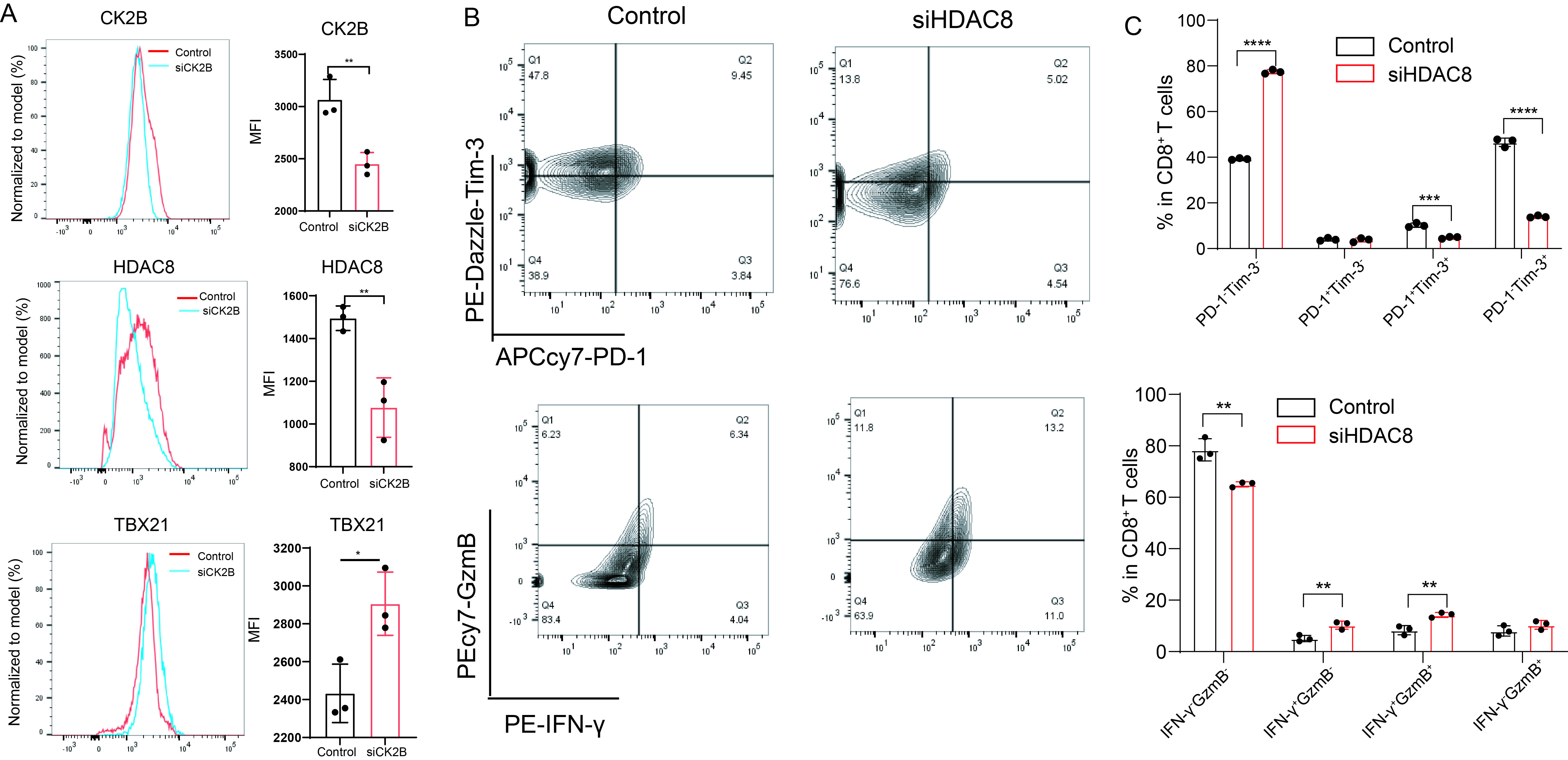


**Fig S11: (A)** Expression of CK2B was knocked down treatment with siRNA in an induced T cell exhaustion model, and flow cytometry determined that the expression of CK2B, HDAC8, and TBX21. **(B)** Typical flow graphs from siHDAC8 and control group. **(C)** Expression levels of PD-1 and Tim-3, as well as IFN-γ and GzmB in CD8^+^ T cells were determined by flow cytometry treatment with siHDAC8 in an induced T cell exhaustion model. **P* < 0.05, ***P* < 0.01, ****P* < 0.001, and *****P* < 0.0001 (one-way ANOVA or Student's t test).


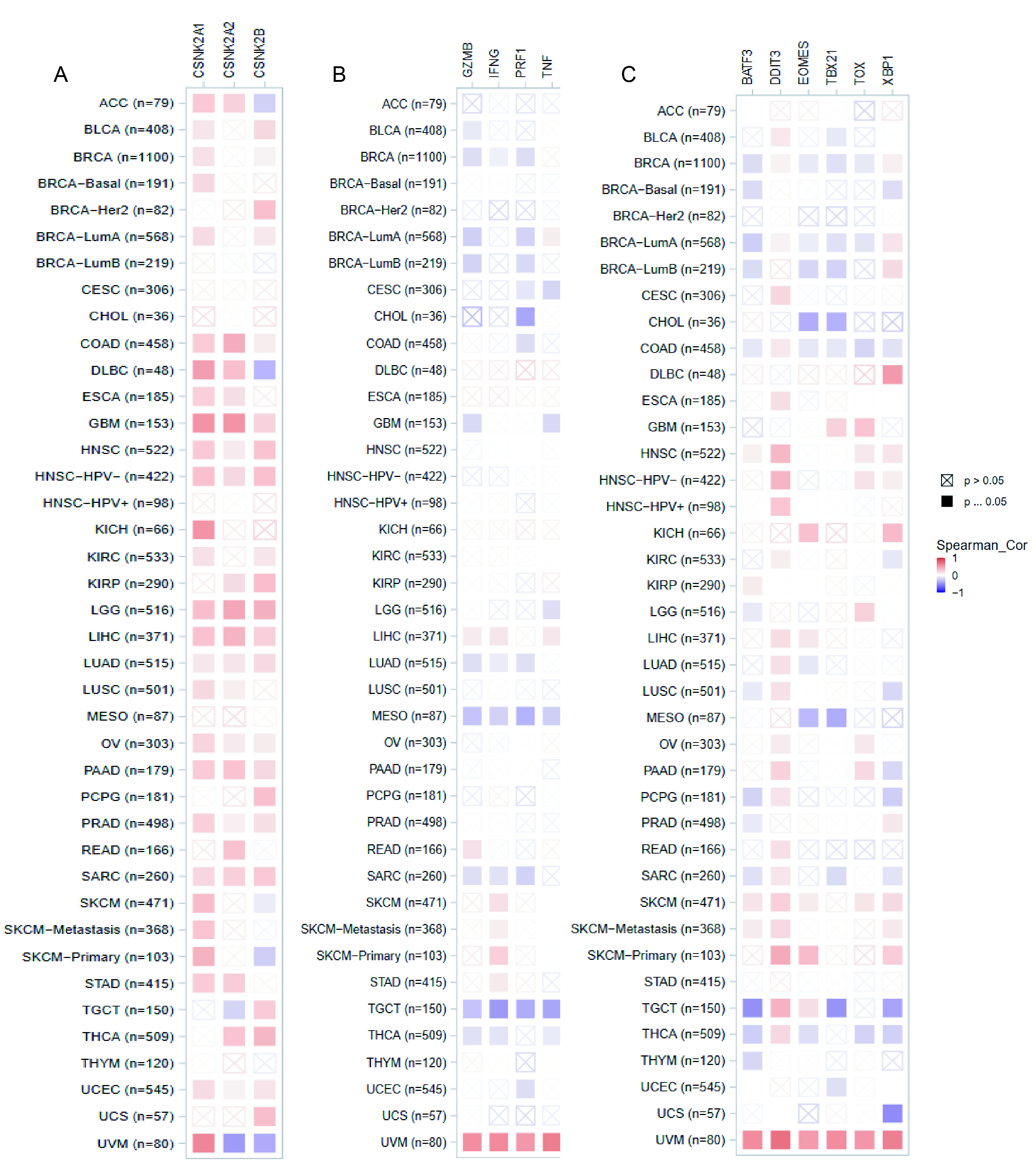


**Fig S12:** **(A)** Public database TIMER 2.0 was used to analyze the correlation between *HDAC8* expression and CK2 family related genes. **(B-C)** Correlation of *HDVC8* expression with cytokines and transcription factors in the TIMER2.0 database.


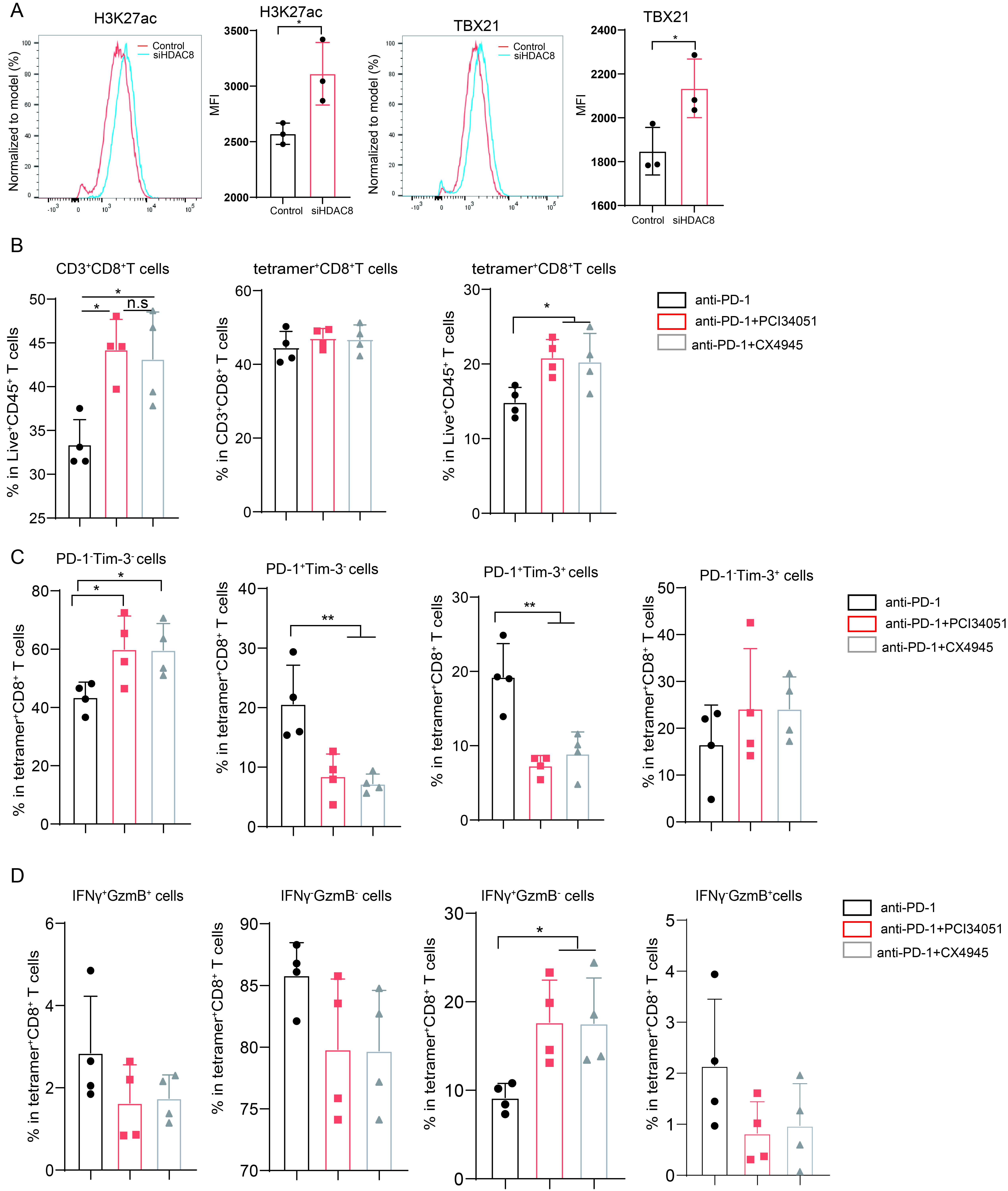


**Fig S13: (A)** Expression of H3K27ac and TBX21 were detected by flow cytometry. Subcutaneous growth of tumor cells (LLC-OVA) in each group of mice treated with PCI-34051 (200 μg/per mouse), anti-PD-1 mAbs (150 μg/per mouse), CX4945 (10 mg/kg), CX4945 plus anti-PD-1 mAbs, and PCI-34051 plus anti-PD-1 mAbs. (**B**) Flow cytometry analysis of the percentage of CD3^+^CD8^+^ T cells and tetramer^+^CD8^+^ T cells, in CD3^+^CD8^+^T cells or live^+^CD45^+^ cells from each treatment groups (n = 4). Flow cytometry analysis of the expression levels of PD-1 and Tim-3 (**C)**, as well as IFN-γ and GzmB (**D**), in tetramer^+^ CD8^+^ T cells from each treatment groups (n =4). **P* < 0.05, ***P* < 0.01, ****P* < 0.001, and *****P* < 0.0001 (one-way ANOVA or Student's t test).

**Table S1: The clinical characteristics of 10 NSCLC patients receiving CIK cell therapy in combination with anti-PD-1 mAbs plus chemotherapy.**

| Characteristics |  | ALL (n = 10) |
| --- | --- | --- |
| Genders | Male | 9 |
|  | Female | 1 |
| Age | >65 | 4 |
|  | ≤65 | 6 |
| Pathological | Adenocarcinoma | 6 |
|  | Squamous carcinoma | 4 |
| Smoke | Yes | 9 |
|  | No | 1 |
| T | T1 | 1 |
|  | T2 | 1 |
|  | T3 | 4 |
|  | T4 | 4 |
|  | N0 | 1 |
|  | N1 | 0 |
|  | N2 | 2 |
|  | N3 | 7 |
|  | M0 | 1 |
|  | M1a | 1 |
|  | M1b | 0 |
|  | M1c | 8 |
| Clinical stage | IIIB | 1 |
|  | IV | 9 |
| Optimal efficacy | PD | 1 |
|  | SD | 3 |
|  | PR | 4 |
|  | CR | 2 |
